# Morphological and molecular data show no evidence of the proposed replacement of endemic *Pomphorhynchus tereticollis* by invasive *P*. *laevis* in salmonids in southern Germany

**DOI:** 10.1371/journal.pone.0234116

**Published:** 2020-06-16

**Authors:** Albert F. H. Ros, Timo Basen, Ruben J. Teschner, Alexander Brinker

**Affiliations:** Fisheries Research Station Baden-Württemberg, LAZBW, Langenargen, Germany; Natural History Museum of London, UNITED KINGDOM

## Abstract

Changes in parasite communities might result in new host-parasite dynamics and may threaten local fish populations. This phenomenon has been suggested for acanthocephalan parasites in the river Rhine and Danube where the species *Pomphorhynchus tereticollis* is becoming replaced by the Ponto-Caspian *P*. *laevis*. Developing knowledge on morphologic, genetic and behavioural differences between such species is important to follow such changes. However, disagreements on the current phylogeny of these two acanthocephalan species are producing conflicts that is affecting their correct identification. This study is offering a clearer morphological and genetic distinction between these two species. As *P*. *tereticollis* is found in rhithral tributaries of the Rhine, it was questioned whether the local salmonid populations were hosts for this species and whether *P*. *laevis* was expanding into the Rhine watershed as well. In order to test for this, brown trout, *Salmo trutta*, and grayling, *Thymallus thymallus* from South-Western Germany watersheds have been samples and screened for the occurrence of acanthocephalan parasites. For the first time, both species were confirmed to be hosts for *P*. *tereticollis* in continental Europe. *P*. *tereticollis* was found to be common, whereas *P*. *leavis* was found only at a single location in the Danube. This pattern suggest either that the expansion of *P*. *laevis* through salmonid hosts into rhithral rivers has not yet occurred, or that not yet ascertained biotic or abiotic features of rhithral rivers hinder *P*. *laevis* to spread into these areas.

## Introduction

Most parasitic organisms are in a co-evolutionary arms race with their host species [1,2]. In horizontally transmitted endoparasites this generally results in relatively low parasitic virulence for the parasites’ adult stages in the final host [3]. However, in punctuated events parasites may reach new host populations or get access to a new host species. The lack of co-evolved mechanisms between these parasites and the new host may potentially lead to elevated virulence resulting in increased host mortality thereby disturbing the equilibrium in the local ecological networks and communities [4–6], as it has been found for the Asian swim bladder nematode *Anguillicoloides crassus* colonizing the European eel (*Anguilla anguilla*) [6]. Therefore, it is important to detect and follow changes in parasite distributions. Records in historical literature are important to evaluate how uncommon such changes are, and such records are mostly based on morphological classification of the parasite species. As molecular methods (barcoding, next-generation sequencing) become more and more accessible to classify species [7,8], it becomes increasingly important to validate the reliability of the different classification methods and how the derived taxonomies relate to each other [9–12].

Acanthocephalans are a small phylum of obligatory endoparasites with limited variation in morphological structures that are common in aquatic ecosystems [13]. They require an arthropod intermediary host, and a vertebrate final host to which they may accrue considerable damage to the intestines [13,14]. Recent literature on Western-European acanthocephalans of the genus *Pomphorhynchus* has shown the importance of complementing morphological with molecular bar-coding methods in order to study recent changes in species distributions [4,15–17]. The current data suggests that Ponto-Caspian lineages of *Pomphorhynchus laevis* spread via the Danube-Main-Rhine canal into Western-Europe [18], and are effectively replacing *Pomphorhynchus tereticollis* populations in the Rhine [15]. Such a replacement could result in increased mortality of final host species as was shown for *A*. *crassus* [6]. The genus *Pomphorhynchus* include 28 species distributed over all continents and six of which have been described from the European continent (Retrieved 13 April 2020, from the Integrated Taxonomic Information System on-line database, http://www.itis.gov). They comprise about 2% of all acanthocephalans which in general are known to infect a wide range of intermediary and final host species to complete their complex life cycles [13,19]. When eggs are ingested by a suitable arthropod as intermediary host, the larva emerges and passes through the gut wall to reach the arthropod’s hemocoel where it further develops into a dormant larval stage, called cystacanth. Such infections are known to change the behaviour of the arthropod increasing the transmission probability of the cystacanth to the final host, usually through predation by a fish [20–22]. With the fish host, the young parasite emerges and uses its proboscis to penetrate the intestinal wall where it attaches, matures and reproduces dioeciously. Eggs produced by gravid females are excreted with the fish’s faeces. Fish occasionally are paratenic with the acanthocephalans remaining in a cystacanth stage in the body cavity [4,19]. The parasites has been shown to cause some inflammation and alteration of the intestinal wall, but this does not result in significant effects on mortality, growth or reproduction of their fish host [14].

Classical studies on this genus have differently addressed the morphological features of the proboscis to categorize and identify *Pomphorhynchus* species [23–25]. Although different forms of *Pomphorhynchidae* have been described in Western Europe, these forms were often combined under the "*P*. *laevis* species-complex" (Zoega in Müller, 1776) [23]. Genetic barcoding based on sequencing parts of relatively well conserved areas of mitochondrial (e.g. COI = cytochrome oxidase subunit I gene) and ribosomal (ITS = internal transcribed spacers) DNA has confirmed that at least one of the forms of the *P*. *laevis* species-complex, that was originally described on morphological characteristics as *P*. *tereticollis* (Rudolphi, 1809), should be considered a distinct species [26]. Dating techniques based on the molecular clock estimate that these two species started to diverge from the Late Miocene, about 8.4 million years ago [27]. Variation in mitochondrial COI and ribosomal ITS DNA further suggest that geographical populations of *P*. *laevis* diverged gradually since the late Miocene leading to genetically [16,28] and morphologically distinct lineages [16], whereas genetic variation in *P*. *tereticollis* is low, with populations starting to diverge from Middle Pleistocene [28].

The relation between the morphological and genetic classification of *P*. *laevis* and *P*. *tereticollis* has recently been questioned again, as molecular and morphological data seemingly pointed to a different species classification [29,30]. This has consequences for the interpretation of distribution patterns of the two species. For example, it was proposed that invasive intermediary and final hosts of Ponto-Caspian origin (e.g. the gammarid *Dikerogammarus villosus* and the round goby, *Neogobius melanostomus*), could carry around their parasites and thus spread non-native *Pomphorhynchus* species to new environments in the Rhine [4]. Identification based on morphological characteristics of parasites from invasive and local hosts, first led to the conclusion that *P*. *tereticollis* would replace endemic *P*. *laevis* [4]. However, a more recent molecular analysis of parasites taken from the invasive fish (*N*. *melanostomus*) indicated that these had ribosomal (ITS) sequences that were similar to those of *P*. *laevis* [29]. Moreover, the morphological characteristics (i.e. hooks of the bulbus) that were used to distinguish *P*. *tereticollis* from *P*. *laevis* [4,26], now also have been found in a subpopulation of these parasites in the Danube (i.e. *P*. *bosniacus*) that is genetically identical to *P*. *laevis* [16].

*P*. *tereticollis* was originally described on morphological characteristics from a specimen collected from European flounder (*Platichthys flesus*) from the Baltic coast [26]. *P*. *tereticollis* has since been verified using bar-coding methods in a wide range of gammarids, cyprinid and perciform hosts in lowland rivers in Europe [19,26]. Also gammarids captured from rhithral rivers in Europe have been shown to be infected with *P*. *tereticollis*, but the fish hosts was not identified in these rivers [31]. In rhithral rivers the dominant fish species are brown trout (*Salmo trutta*) and grayling (*Thymallus thymallus*). Trout and other salmonids are known to be suitable fish hosts for *Pomphorhynchus* species in the British Islands [24,32]. Although *P*. *laevis and P*. *tereticollis* have been collected from brown trout on the British Islands and Ireland [28], to the best of our knowledge salmonids have not been described as fish hosts for *P*. *tereticollis* in continental Western Europe.

As Ponto-Caspian lineages of *P*. *laevis* are spreading in the Rhine and Danube, it was questioned whether these parasites could have spread upstream in mountain rivers in Baden-Württemberg to infect Salmonidae. We expected *P*. *tereticollis* to be endemic as it has been commonly found in gammarids in the region in tributaries of the Rhine (in neighbouring Switzerland) [31]. The aims of this study, which was carried out in upstream tributaries of both Rhine and Danube in the federal state of Baden-Württemberg, were threefold: 1) to describe relationship between morphological and molecular data for the local *Pomphorhynchus* species; 2) to answer whether salmonids, e.g. brown trout and grayling, might serve as fish hosts for these parasites in Baden-Württemberg, and 3) to establish whether Ponto-Caspian lineages of *P*. *laevis* have expanded from the Rhine into rhithral rivers.

## Material and methods

### Ethics statement

Approval of our present study by a review board institution or ethics committee was not necessary because all fish were caught under the permission of the local fisheries administration and all needed qualifications for the involved people (fishing licenses) were checked regularly by the local member of the animal protection committee. Electrofishing was conducted under a license from the fisheries administration (Regierungspräsidien Baden-Württemberg) after informing the local authorities, and under general allowance granted to the Fischereiforschungsstelle (§ 6 and § 22 of the "Landesfischereiverordnung" law of the state Baden-Württemberg). Fish were stunned by a blow to the head and expertly killed immediately by a cardiac stab according to the German Animal Protection Law (§ 4) and the ordinance of slaughter and killing of animals (Tierschlachtverordnung § 13). No living fish were used.

### Sampling

**Fig 1:**
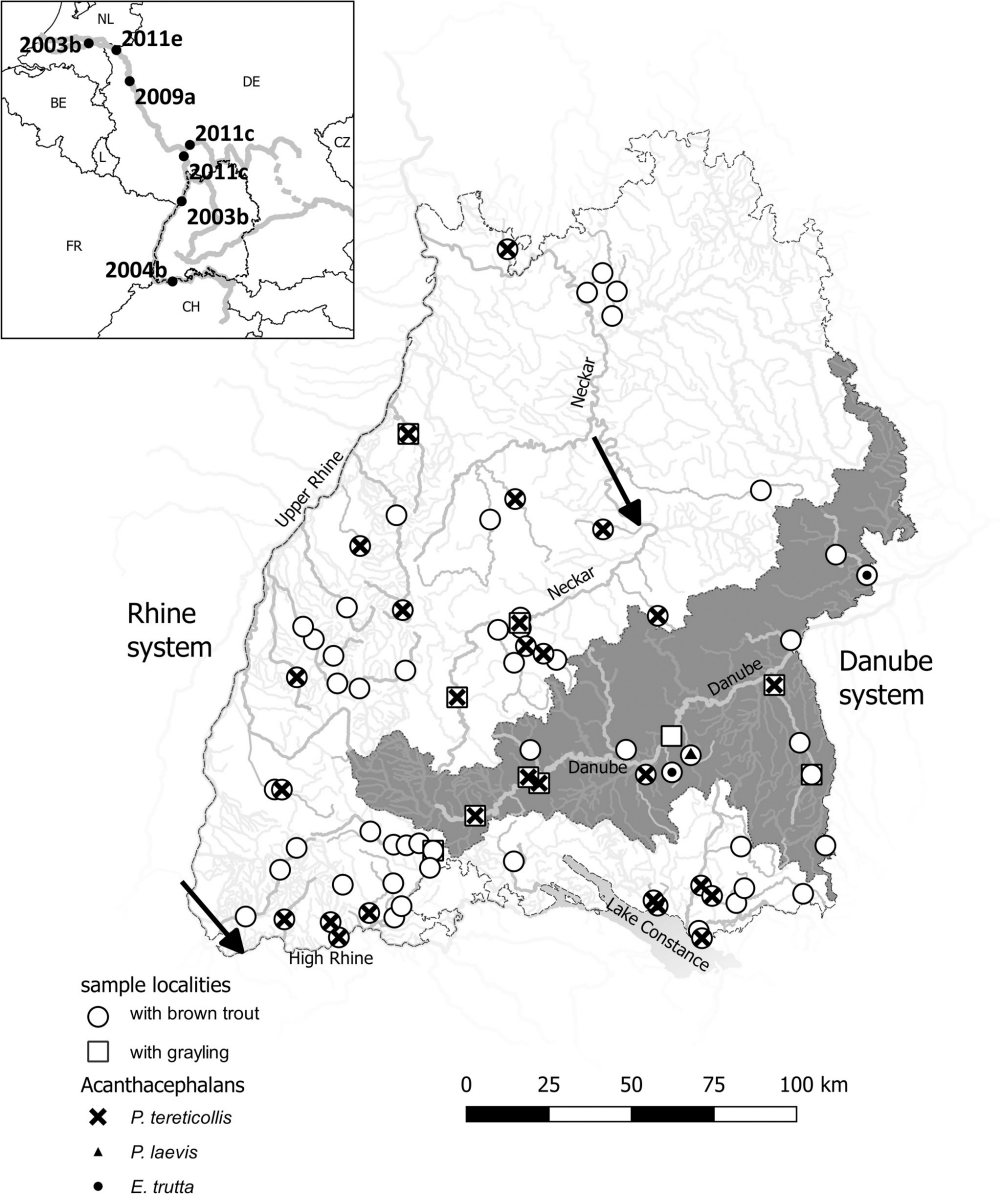
Acanthocephalans in Baden-Württemberg. Sample localities in the Rhine (white area) and Danube (grey area) systems in Baden-Württemberg, Germany that were checked for acanthocephalan species. Circles represent sample localities with brown trout, squares represent sample localities with grayling; black crosses: *Pomphorhynchus tereticollis*; black triangles: *P*. *laevis*; black points: *Echinorhynchus trutta*. The insert overview shows year and localities on the Rhine and Neckar where in the literature *P*. *laevis* was reported [a: 4, b: 15, c: 29, d: 35, e: 36]. The two arrows show the most southern distribution in Rhine and Neckar with round gobies in the period until 2017 [37]. Gis shapes were provided under (CC) by LGL (https://www.lgl-bw.de) and FFS (https://lazbw.landwirtschaft-bw.de/pb/,Lde/Startseite/Themen/Fischereiforschungsstelle).

In the summer of 2017 and 2018, young (sub-yearling to yearling) brown trout (n = 588) and grayling (n = 63) were sampled as part of an ongoing study at the Fischereiforschungstelle (Langenargen, Germany) of a salmonid disease (“proliferative kidney disease”) in Baden-Württemberg, Southern Germany. The localities were part of 8 rivers that flow into Lake Constance, 16 tributaries of the Rhine, 11 tributaries of the Neckar, 9 tributaries of the Danube, and the start of the Neckar and Danube rivers (Fig 1, S1 Table). All samples were taken from summer-cold rhithral river sections. These sections have been classified based on their historical fish assemblages, and have associated physico-chemical and hydromorphological quality elements to support their ecological status in the framework of the European Water Framework Directive, and the German ordinance on the protection of surface waters [33,34]. Table 2 provides the codes of these rivers. Those waters exhibit summer temperatures not rising above 20–23°C with high oxygen levels near 100% satiation. Some larger lowland rivers were sampled to search for *P*. *laevis*. These rivers are may reach maximum summer temperatures of 28°C [33].

Fish were captured using electrofishing and expertly killed immediately (see Ethics statement). Then either their intestines were directly dissected, and parasites kept on RNAlater (Invitrogen), or fish were frozen (-80°C) for later preparation of the intestines to check for acanthocephalan parasites.

### Morphological characterization of acanthocephalans

**Fig 2:**
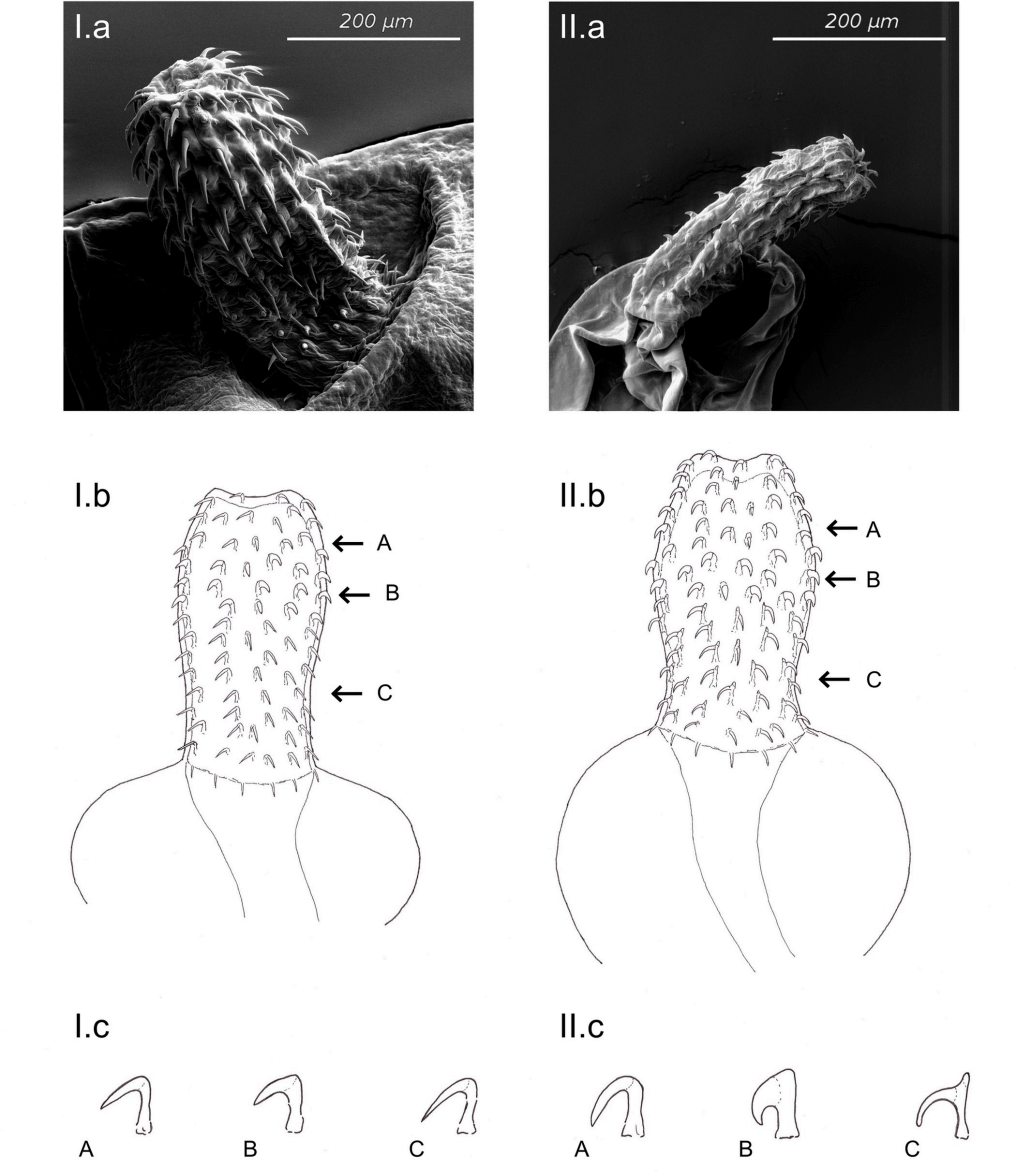
Morphology of Pomphorhynchidae in Baden-Württemberg. Scanning electron microscopy images (a), and scientific drawings of the proboscis and bulbus (b) and hooks (c) of: (I) *P*. *laevis*, and (II) *P*. *tereticollis*. The following characteristics were different between *P*. *tereticollis* and *P*. *laevis* in Baden-Württemberg: 1) larger variation in hook shape from tip of the proboscis to bulbus (A *vs*. B *vs*. C) in *P*. *tereticollis*; 2) hooks on position 5–6 counted from tip of the proboscis to bulbus (B) are clearly more robust in *P*. *tereticollis*; 3) hooks of *P*. *tereticollis* closest to the bulbus (II.c.C) have a projection, in *P*. *laevis* this projection is missing (I.c.C).

A first classification of *Pomphorhynchus* individuals was done based on the shape and position of the hooks [16,26] using light microscopy (Leica DMLS). The proboscis was dissected and squashed on a microscope slide and evaluated under 500x to 1000x magnification. For a more precise description of the differences in habitus of the two species scanning electron microscope images were made (Fig 2). Parasites fixed in 70% ethanol were washed in water and then mounted on a specimen stub. After overnight air drying, the parasites were sputter-coated with palladium-gold (Emitech K550) and then scanned on a Zeiss DSM 960A. Based on these light and scanning electron microscopy images a scientific drawing was made exemplifying the differences that were used to morphologically distinguish between the species (Fig 2).

### Molecular methods

Molecular methods were used to support the classification based on morphological differences. DNA was extracted from single acanthocephalans following a published CTAB (cetyltrimethylammonium bromide) based protocol [28]. In order to genetically barcode the specimens two approaches were used: 1) a quick screening qPCR protocol was developed based on the published COI gene variation [27]; 2) Barcoding using Sanger sequencing of COI and ITS template for a selection of the parasite extracts. For the first method (Table 1), three internal primer pairs were designed using NCBI/primer-BLAST [38]: Primer-T1) specific for *P*. *tereticollis* lineage Pt_L2/L3 [27]; Primer-T2) generally specific for *P*. *tereticollis*; and Primer-L) specific for *P*. *laevis* Pl_L1 [27]. A standard qPCR (GoTaq®, Promega, A6001) protocol was carried with BRYT Green® as the DNA-intercalating reporter dye. A maximum of 25 cycles was set as after 26 cycles some primer dimer activity became noticeable. In each qPCR run extracts of *P*. *laevis* and *P*. *tereticollis* were added as positive and negative control.

**Table 1:** Primer sequences, melting temperatures (Tm), and base length.

Primer name	Sequence (5'->3')	Tm °C	Length	Ref.
PL-fw	TGACTCATGCCAGTGATGTTAG	58.4	22	designed
PL-rev	AAGCAAGGACACACCTATAACC	58.4	22	designed
PT1-fw	ATAGTGACAACTGCGGGATTAG	58.4	22	designed
PT1-rev	TGGAGTTCAAATTACGGTCCAT	56.5	22	designed
PT2-fw	CCTCATGTTGAGGGATTACAGG	60.3	22	designed
PT2-rev	TGCCACCCAAGTAACCAAA	54.5	19	designed
LCO1490	GGTCAACAAATCATAAAGATATTGG	56.4	25	[28]
HCO2198	TAAACTTCAGGGTGACCAAAAAATCA	58.5	26	[28]
ITS_BD1	GTCGTAACAAGGTTTCCGTA	55.3	20	[28]
ITS_BD2	TATGCTTAAATTCAGCGGGT	53.2	20	[28]

For the second method general primers for COI mDNA (LCOI1490, HCOI2198) and ITS rDNA (BD1, BD2) were used (Table 1) following the method of Perrot-Minnot [28]. PCR (QUIAGEN) products were visualized by agarose gel electrophoresis. A single band was cut out of the gel, and of the cleaned DNA product two aliquots, one mixed with the forward primer and one mixed with the reverse primer, were sent to Eurofins Genomics TubeSeq service (Sequencing Lab Cologne, Eurofins Genomics, Köln, Germany) for sequencing. Sequences have been deposited in the Barcode of Life Data System. (code POBW, www.boldsystems.org) and on GenBank® (http://www.ncbi.nlm.nih.gov/genbank/): accession numbers: MT216136- MT216144 and MT216149-MT216172.

### Data management and analysis

The sequences of forward and reverse amplicons were manually aligned, and analysed using the MEGA-X software (version 10, http://www.megasoftware.net). Following the methods in David *et al*. [18] sequences were compared with publicly available sequences from GenBank: accession numbers for the COI sequence: LN994840 to LN994950 for *P*. *laevis* [27]; MK612497 to MK612423 for *P*. *bosniacus* [16]; LN994950 to LN994994 for *P*. *tereticollis* [27]; DQ089710 and KP261013 for *E*. *truttae* [39,40]; KF156892 for *E*. *gadi* [41]; accession numbers for the ITS sequence: AY135415 to AY135417, AY424669, KF559305 to KF559307, KJ756498 to KJ756500, MH319898, MH319899, MK157040, MK157041 for *P*. *laevis* [17,28,29,42,43]; AY424670, KY075791 to KY075817 for *P*. *tereticollis* [28,44]; EF107643 to EF107648 for *E*. *gadi* [45]. To simplify the comparison with published sequences first consensus sequences were calculated based on available information on genetic lineage and sample area (R 3.6 msa package, see S3 Table). Then the most likely evolutionary relationships between samples and with related samples from GenBank, were inferred using the Minimum Evolution method [46,47], calculating evolutionary distances using Maximum Composite Likelihood [48]. Genetic distances (*d*) between and within groups were based on the Kimura 2-parameter model that distinguishes between transitions (changes within pyrimidines or purines) and transversions (changes between pyrimidines and purines) and rates among sites were assumed to be Gamma distributed.

Statistical analyses were carried out using JMP® PRO (version 14.2.0 (64-Bit), SAS Institute Inc.). To measure the correspondence between identification methods (qPCR vs microscopy) we estimated a 95% confidence interval range based on a binomial distribution of outcomes (tests in which both methods agree vs. total number of tests carried out). In order to test differences in the prevalence of parasite infections between drainage areas the Pearson’s Chi-squared test was used. The parasite intensity (intensity) was tested using a standard least squares (linear) model (LM) on square root transformed data with waterbody (4 levels: Bodensee, Rhine, Neckar and Danube), fish host species (2 levels: brown trout and grayling) and fish host total length as explaining variables, using river as a random factor:

$$LM(\sqrt{intensity}∼waterbody+host:\;(species\times total\;length)+random(river))$$

## Results

### Morphological identification

**Fig 3:**
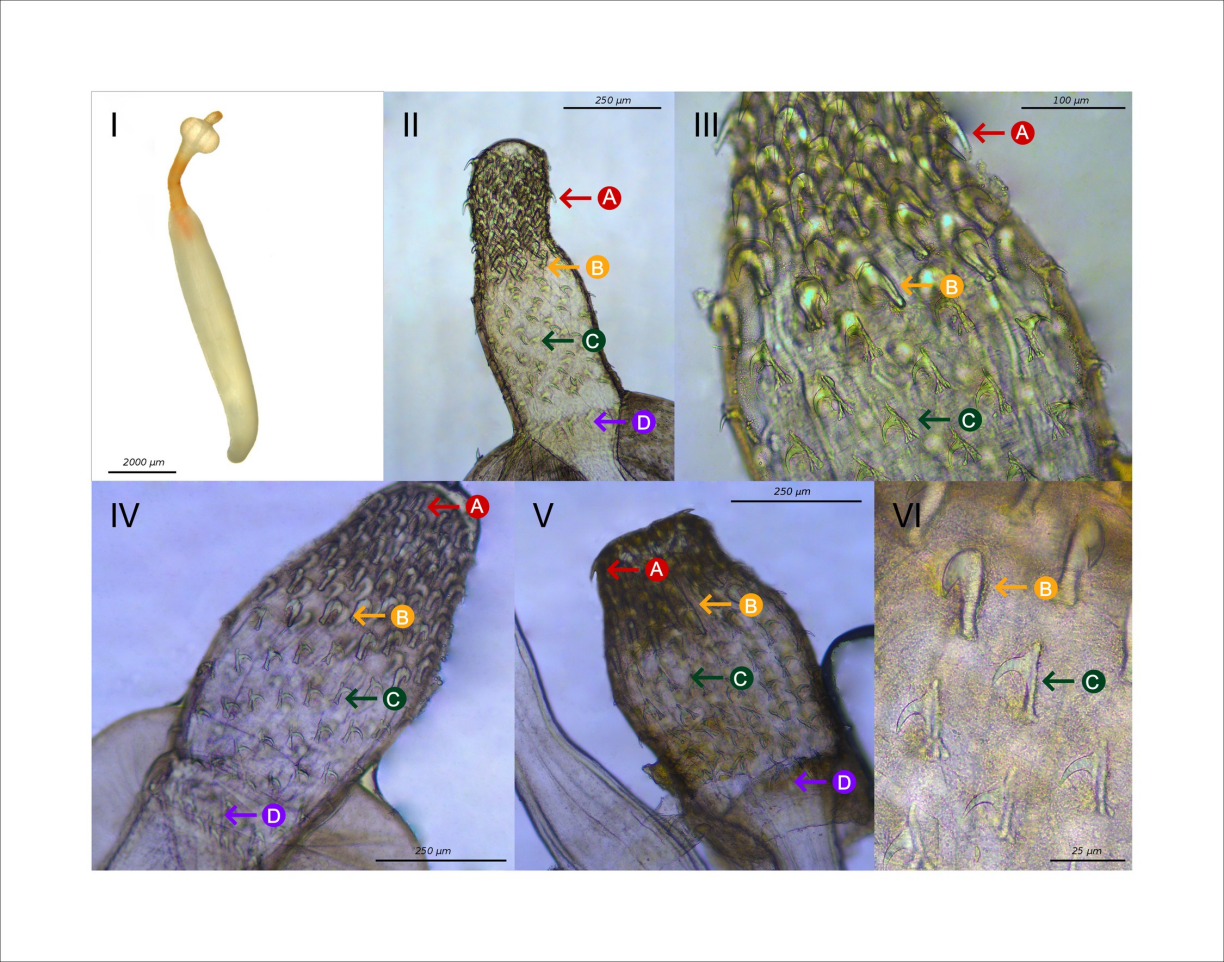
I) Habitus of *P*. *tereticollis*. II-VI) Close up of the proboscis. Clearly visible are: A) simple hooks at the first (1–5) positions B) the stout hooks at the middle (5–6) positions; C) the hooks from position 7 onwards with the defining proximal structure on the top; D) a ring of hooks on the bulbus.

In total 588 brown trout (mean total length: 10.9 cm, range 6.0–24.0 cm) and 63 grayling (mean total length: 13.4 cm, range 6.7–22.2 cm) were screened for intestinal acanthocephalans (Table 2). Based on morphological differences of the proboscis, 120 individuals, 104 in brown trout and 16 in grayling, were classified as *Pomphorhynchus tereticollis* (Fig 2). One specimen was identified as *Pomphorhynchus laevis*, while four specimens did not have a bulbus and were identified as *Echinorhynchus spec*. Both *P*. *laevis* and *Echinorhynchus* specimens originated from samples of tributaries of the Danube (Fig 1). The morphological differences used to classify the *Pomphorhynchus* species were the following: 1) *P*. *tereticollis* has bulkier hooks in the middle rows (position 5–6 counted from the top) of the proboscis while in *P*. *laevis* there is less variation in hook size (Figs 2 and 3: compare feature B with A and C); 2) *P*. *tereticollis* has a noticeable extension at the top of the hooks further to the base of the proboscis, which lacks in *P*. *laevis* (Figs 2 and 3: feature C); 3) *P*. *tereticollis* has a ring of hooks on the bulbus, which lacks in *P*. *laevis* (Fig 3: feature D).

**Table 2:** Distribution of acanthocephalans over sampled types of rhithral rivers. Classification was based on morphological characteristics; with for 25 capture sites at least one individual checked using molecular methods (see S4 Table).

Species	river type	*P*. *tereticollis*	*P*. *laevis*	*E*. *trutta*	Fish total
brown trout	Cyp-R	23	1	0	94
	Sa-HR	35	0	4	174
	Sa-MR	28	0	0	196
	Sa-ER	18	0	0	124
grayling	Cyp-R	16	0	0	52
	Sa-MR	0	0	0	1
	Sa-ER	0	0	0	10
Total		120	1	4	651

River type codes: Cyp-R: lower river section where cyprinids and salmonids are found together; Sa-HR: hyporhithral = lower rhithral section; Sa-MR: metarhithral = middle rhithral section; Sa-ER: epirhithral = higher rhithral section. In Sa-HR, Sa-MR and Sa-ER salmonids are the dominant species. For classification used see OGewV [33].

### Prevalence and intensity

The prevalence of *P*. *tereticollis* infections did not show a significant difference between grayling and brown trout, being 25.4% of 63 and 17.7% of 588, respectively (χ^2^ = 2.25, df = 1, p = 0.13). Overall, the highest parasite prevalence was in rivers flowing in Lake Constance, with 33.9% of sampled fish being infected (n = 115). The prevalences in tributaries of the river Rhine, Neckar and Danube were significantly lower (χ^2^ = 22.7, df = 3, p < 0.001) than that of the Lake Constance area, ranging from 13.3% in the Neckar (128 fish) to 15.4% in the Rhine (285 fish) and 16.3% in the Danube (123 fish) area.

The intensity of *P*. *tereticollis* infection ranged between 1 and 15 individuals with a mean of three individuals. Larger animals tended to have more *P*. *tereticollis* (LM with river as random effect, positive relation with total length: F(1,106) = 10.7, p < 0.01), independent of species or drainage area (all p > 0.43). The single individual of brown trout infected with *P*. *laevis* had 4 individuals, and number of *E*. *trutta* in the four infected brown trout ranged from one to three individuals.

### Molecular analysis of the acanthocephalan lineages

Of each capture site with parasites (n = 31) at least one individual was sampled for molecular identification using a qualitative positive/negative qPCR test. This test based on selected “internal-primer” for *P*. *tereticollis* and *P*. *laevis* almost invariably supported the morphological classification of the specimens (Table 3, S4 Table). Because few specimens of *P*. *laevis* were detected in the studied salmonids, seven additional chub (*Squalius cephalus*) were checked for Pomphorhynchidae in three different localities (Argen, Nonnenbach and Kinzig, S4 Table). These animals have been added for molecular parasite identification. For primers L a positive quantification (start of exponential phase) was reached at a mean cycle number of 11.8 (range: 9.0–17.4), with all negative quantifications being higher than 25. For primers P1 positive quantification was reached at CT = 14.5 (range: 11.0–17.6) with CT values of negative samples ranging from 23 to 25, and primers P2 had positive quantification at CT = 13.7 (range: 11–16.7) and negatives ranging from 19.7 to 25. Late duplication in the negative P2 samples was possibly due to some primer-dimer activity or non-specific binding. In 38 out of 41 cases the qPCR gave a clear distinction between P and L that confirmed the morphological classification (Table 3). In one case, morphologically classified as *P*. *tereticollis*, all primers were positive (CT values: L: 17.7 > P1: 14.3 and P2:12.9). In two cases all primers tested negative. In these two latter cases the proboscis of these two individuals were missing a bulbus, and thus were morphologically clearly different of *Pomphorhynchidae* (i.e. *Echinorhynchus spec*.). Thus overall, only one out of 39 measurements was inconclusive (95% confidence interval of the association between both measurements = 85–99%, binomial test).

**Table 3:** Association of microscopy and a qualitative qPCR method using nested primers.

qPCR amplification				acanthocephalan species based on microscopy			
PL	PT1	PT2	qPCR result	*P*. *laevis*	*P*. *tereticollis*	*E*. *spec*.	fish host species
+	-	-	*P*. *laevis*	4	0	0	1 brown trout + 3 chub
-	+	+	*P*. *tereticollis*	0	34	0	4 grayling, + 27 brown trout + 4 chub
-	-	-	not *P*. *tereticollis* not *P*. *laevis*	0	0	2	2 brown trout
+	+	+	*P*. *tereticollis*? *P*. *laevis*?	0	1*	0	1 brown trout
			total	4	35	2	41 samples

Primer PL anneals to Western European COI sequences of *P*. *laevis*; primer PT1 and PT2 anneal to COI sequences of *P*. *tereticollis*. Amplification result: + = positive,— = negative. * = the record with inconclusive qPCR results. For raw data see S4 Table.

Sanger sequencing of the COI gene resulted in an amplicon length of 663bp for all species (“between primer sequence” of 612bp) (*e*.*g*. S2 Table). Amplicon length for the ITS sequences were more variable. One ITS sequence was obtained for *P*. *laevis* with a length of 662bp (+40bp for primers). ITS sequences were 690bp (+40bp for primers) for *P*. *tereticollis* (S2 Table 3.3). Within the 14 COI sequences of *P*. *tereticollis* 13 different haplotypes were detected. Of the 11 ITS sequences of *P*. *tereticollis* four haplotypes were detected with 2 of them shared between many individuals (resp. 4 and 5 individuals). Within group genetic distance for *P*. *tereticollis* in Baden-Württemberg were 0.0066±0.0021 (*d* ± S.E.) and 0.0014±0.00056 (*d* ± S.E.) for COI and ITS resp.

Three COI and two ITS sequences were extracted for the *E*. *spec*. individuals that were obtained from brown trout captured from the rivers Schwarzach and Brenz. These were clearly deviating from *Pomphorhynchus* (S1A and S1C Fig, and S2 Table). The 612bp “between primer sequences” for COI had a >99% identity with published *E*. *truttae* sequences (S1A Fig). Mean genetic distance with these *E*. *truttae* sequences, was 0.0053±0.0028 (d±S.E). Genetic distances with one of the *Pomphorhynchus* lineages and species was ranging from 0.47 to 0.60. No published ITS sequences of *E*. *truttae* were available for comparison. The closest match with the obtained 564 and 576bp sequences was that of *E*. *gadi* (S1C Fig).

A test of evolutionary relationships between the samples using the minimum evolution method (S1A and S1C Fig) showed a strong resemblance of COI and ITS sequences of the *P*. *laevis* samples from Baden-Württemberg with published *P*. *laevis* sequences belonging to Western-European lineages. The *P*. *tereticollis* lineages showed little variation in COI and ITS sequences (S1B and S1C Fig: bootstrap confidence for separate branches < 91%). Lowest genetic distances for COI sequences between the samples taken in Baden-Württemberg samples and published samples were found for the Western European and Ponto-Caspian European Pt-L2 and Pt_L3 lineages [27]: i.e. 0.0044±0.0014 and 0.0096±0.0033 resp. (*d* ± S.E.). The genetic distance with the Pt-L1 lineage was 0.020±0.005 (*d* ± S.E.). The COI gene sequences of the *P*. *laevis* that were extracted from a chub in the Rhine area (river Kinzig) and from a brown trout in the Danube area (river Kanzach) were highly similar, showing only 3 transitions in the 663 bp amplicon length. These *P*. *laevis* were related to the Western European lineage (S1A Fig, [lineage Pl_L2:, 27]). Genetic distance of the Baden-Württemberg samples with the Western European lineage was 0.027±0.005 (*d* ± S.E); with genetic distances ranging from 0.11–0.22 with the other lineages.

## Discussion

The framework of the study is an ongoing invasion of a Ponto-Caspian lineage of the acanthocephalan parasite *Pomphorhynchus laevis* in the Rhine and Danube [15,18]. It was hypothesized that this invasive lineage might migrate (via one of its intermediate or final hosts) further upstream in rhithral rivers in the Rhine and Danube areas, and thereby replaces the endemic *Pomphorhynchus* species which would alter existing parasite-host relationships. Using both morphological and molecular methods, the current study did not detect the invasive Ponto-Caspian lineage of *P*. *laevis* in the 120 samples obtained from rhithral rivers in Baden-Württemberg, Southern Germany. Most specimens found were of the endemic *P*. *tereticollis* and comparison with published sequences of DNA of the mitochondrial cytochrome oxidase subunit 1 (COI) and the ribosomal internal transcribed spacer (ITS) showed that all *P*. *tereticollis* and *P*. *laevis* had little genetic variation within the sampled area and were closely related to Western European lineages of the parasites.

In the current study species were initially classified based on differences in morphology using SEM, scientific drawing and microscopy (Figs 2 and 3). Most parasites that were collected in the study area showed bold hooks in middle rows on the proboscis and a characteristic projection on the top of the lower hooks on the proboscis. As these characteristics have been consistently attributed to specimens of *P*. *tereticollis* [4,15,26] most individuals were initially classified as this species. However, a recent study of Reier *et al*. [16] using both molecular and morphological methods, showed that *Pomphorhynchus* specimens captured in the Austrian part of the Danube with morphological characteristics of the proboscis that would classify them as *P*. *tereticollis*, had gene sequences (COI) that placed them closely related to *P*. *laevis*. In consequence, molecular evidence is necessary to certainly classify specimens: first a rapid qPCR method was developed to distinguish *P*. *laevis* from *P*. *tereticollis* for the populations of parasites in Baden-Württemberg based on 3 internal primers of the COI sequence for *Pomphorhynchus*. Two of these primers were specific for *P*. *tereticollis* and one for *P*. *laevis*. A large sample of the specimens collected confirmed the morphological classification with an accuracy of 85–99% (the lower threshold being caused by a single ambiguous outcome). A subset of these samples was Sanger sequenced (ITS and COI) and comparison with published sequences (Blastn, NCBI) confirmed the species identification.

The results show that fast molecular qPCR methods is effective for accurate molecular identification of local parasite species. Nevertheless, this cannot replace the first morphological assessment of the species. Moreover, a species classification based solely on measures of genetic similarity may fail to detect important variation in morphologic features that could be explanatory to delineate species [7], or may fail to detect life-history relevant variation in morphological features within species [49]. For example, the host environment might affect morphological features as has been shown for differences in hooks in a tapeworm species depending on being collected from perch or pike [49]. It therefore was concluded that both morphology and different molecular “barcoding” techniques (based on both ITS and COI gene sequences) are necessary to understand how morphological and genetic classification characteristics may be superimposed [16] in this intricate group of acanthocephalans.

Most studies on *Pomphorhynchus* species [4,15,18,19,26,27] have been carried out in larger lowland summer warm (potamal) rivers which are rich in fish species and dominated by cyprinid species. In these rivers both *P*. *laevis and P*. *tereticollis* can be found in a wide range of fish hosts in Central (including the Rhine) and in Ponto-Caspian (including the Danube) Europe [15,18,19,26], with cyprinid species like chub (*Squalius cephalus*) and barbel (*Barbus barbus*) as the most common hosts [19]. Here samples were taken from the more elevated, summer-cold and fast-flowing rhithral tributaries of the Danube and Rhine where salmonids are often the most abundant species. These samples clearly show that young brown trout and grayling are hosts for acanthocephalans in Baden-Württemberg. Almost all acanthocephalans that were found in brown trout and grayling in Baden-Württemberg resembled *P*. *tereticollis*. So far it was only shown for brown trout from the British Island to be a hosts for *P*. *tereticollis* [27], while grayling has never been described to be a host for *P*. *tereticollis*. The prevalence of *P*. *tereticollis* was higher in the Rhine area than in the Danube area, and was highest in tributaries of Lake Constance. It remains to be tested whether the Lake Constance plays a role in maintaining the parasite population at this higher level in tributaries of the lake.

No support was found for an invasion of the rhithral with Ponto-Caspian *P*. *laevis*. Only one brown trout in a tributary of the Danube was found infected with *P*. *laevis*. Molecular data indicate that this specimen originated from the Western European lineage of the species, and was clearly distinct of the invasive Ponto-Caspian lineage [18]. Also *P*. *laevis* sampled from chub in the river Kinzig was mostly related to the Western European lineage. There are two possible explanations for this deviating pattern found in “mountain rivers” (rhithral) from the ongoing invasion with Ponto-Caspian lineages of *P*. *laevis* in lowland rivers (potamal):

First, the invasion of *P*. *laevis* in the main lowland rivers connected to the Rhine and Danube is still ongoing, but they have not yet reached the upstream rhithral tributaries of these rivers that were sampled in the current study. *P*. *laevis* in the Rhine was rapidly expanding from 2003, reaching the High Rhine South of Baden-Württemberg already in 2004 [15,18], more than 10 years before the current study (see Fig 1). David *et al*. [18] postulated that *P*. *laevis* haplotypes that before were restricted to Eastern European lowland rivers, have expanded geographically via the Main-Danube canal to the Rhine as parasites of the round goby (*Neogobius melanostomus*). The round goby acts as a paratenic host for the parasite [4], but parasites can further develop and reproduce after predation of gobies by a piscivourous final hosts [4,29]. The front of the upstream migration of the round goby in the Rhine of Baden-Württemberg occurred already around 2011–2014 [50,51] and current census data of the round goby show the species is currently abundant in the Rhine and Neckar [37]. Indeed, Hohenadler *et al*. [15] has shown that *P*. *laevis* is in the process of replacing the endemic *P*. *tereticollis* in eels captured from the High Rhine. However, the gobies did not passed the Rhine Falls to reach the Bodensee area and have also not spread into the river Danube in Baden-Württemberg [37,52]. This is consistent with the front of the expansion of *P*. *laevis* over the Danube not reaching Austria yet in 2006 [18] and our finding that Graylings from the Danube River being infected with *P*. *tereticollis* rather than *P*. *laevis*. Therefore, the expansion of the round goby might explain the missing distribution of *P*. *laevis* in the Danube and Bodensee area, but cannot explain the missing distribution of *P*. *laevis* in the Rhine and Neckar area.

Second, the missing distribution of *P*. *laevis* in tributaries of the Rhine is suggesting that *P*. *laevis* might be less able to establish or maintain in the rhithral than *P*. *tereticollis*. The most likely explanation is that round gobies do not migrate from the Rhine into mountain rivers, thereby limiting further spatial spread of parasites it carries. However, cyprinid species like chub, barbel and nase (*Chondrostoma nasus*) that are final hosts for *P*. *laevis* [19,53], are known even to pass fast flowing barriers between potamal and rhithral rivers in order to reach upstream spawning areas [54–56]. Moreover, strains of brown trout are known to migrate between lowland and rhithral rivers, and hybrids between trout from the Danube and Rhine have been found in the upper Danube basin [57].Thus, both cyprinids and trout might carry the parasites upstream to the rhithral fish populations including the Rhine and Danube populations. Nevertheless mainly *P*. *tereticollis* was found in rhithral fish hosts (young brown trout and grayling) in this study.

Thus, *P*. *tereticollis* seems to own certain properties to successful adapt to the rhithral environment which in *P*. *laevis* are missing. A possible explanation for such a difference is the way these species manipulate their gammarid host in order to reach their final fish host. Recently Perrot-Minnot *et al*. [19] showed that in an area with sympatric occurrences of *P*. *laevis* and *P*. *tereticollis*, chub was more often infected with *P*. *laevis* while barbel was more often infected with *P*. *tereticollis*. They proposed this to be due to a weaker alteration in phototaxis and geotaxis in gammarids infected with *P*. *tereticollis*. As gammarids tent to drift downstream with current and thus have to actively migrate upstream [58], such an alteration in the behaviour of *P*. *laevis* infected gammarids would make them more vulnerable for effects of river current than *P*. *tereticollis* infected gammarids. A larger effect of drifting downstream would make it less likely for *P*. *laevis* infected gammarids to maintain themselves in the fast flowing rhithral zone and thereby to reach salmonids as final hosts. This explanation is consistent with Westram *et al*. [31] who found that *P*. *tereticollis* infected gammarids are abundant in rhithral rivers in Switzerland, while *P*. *laevis* infected gammarids were only found in one locality in Switzerland. It is becoming increasingly evident that environmental factors must be taken into account in understanding the biogeography of parasites or diseases [59]. The accumulated knowledge about the distribution of different *Pomphorhynchus* species, indicate geographical constraints might limit the spread of *P*. *laevis*, even when areas are connected by its intermediate and fish hosts. This implies that salmonids in rhithral rivers would not likely experience a substitution of *Pomphorhynchus* species as took place for eels in the river Rhine [15].

In summary, the current data shows that *P*. *tereticollis*, so far mostly associated with cyprinid fish in lowland (potamal) rivers, is generally able to infect brown trout and grayling, the most abundant salmonid fish species in the rhithral rivers of Baden-Württemberg. The parasite haplotype that was found in Baden-Württemberg Pt-L2/3, has been found in a wide range of fish hosts including the most common, i.e. the barbel and chub, but also in brown trout in the Otter river, UK [27]. To the best of our knowledge the parasite has not been shown yet to infect grayling and brown trout in continental Europe. Due to the possible differential behavioural effects of *P*. *laevis* and *P*. *tereticollis* on gammarids [19] in combination with the wandering of Salmonids, rhithral areas may provide a refuge for *P*. *tereticollis* when alien *P*. *laevis* lineages are expanding.

## Supporting information

S1 Fig Evolutionary relationships. (DOCX)

S1 Table Sample sites and prevalence in brown trout (*Salmo trutta*) and grayling (*Thymallus thymallus*) in Baden-Württemberg during the survey in 2018–2019. (DOCX)

S2 Table Examples of Sanger sequences of acanthocephalans in Baden-Württemberg. (DOCX)

S3 Table Consensus sequences used for genetic analyses. (DOCX)

S4 Table qPCR results supporting the morphological characterization. (DOCX)

S1 Appendix Statistical result. (DOC)
