# Supplementary material for: Morphological and molecular data show no evidence of the proposed replacement of endemic Pomphorhynchus tereticollis by invasive P. laevis in salmonids in southern Germany
Source: PLoS One. 2020 Jun 16;15(6):e0234116. doi: 10.1371/journal.pone.0234116 (PMC7297375; doi:10.1371/journal.pone.0234116)
Supplement: S1 Fig — (DOCX) [file pone.0234116.s001.docx]

**S1 Fig. Evolutionary relationships**


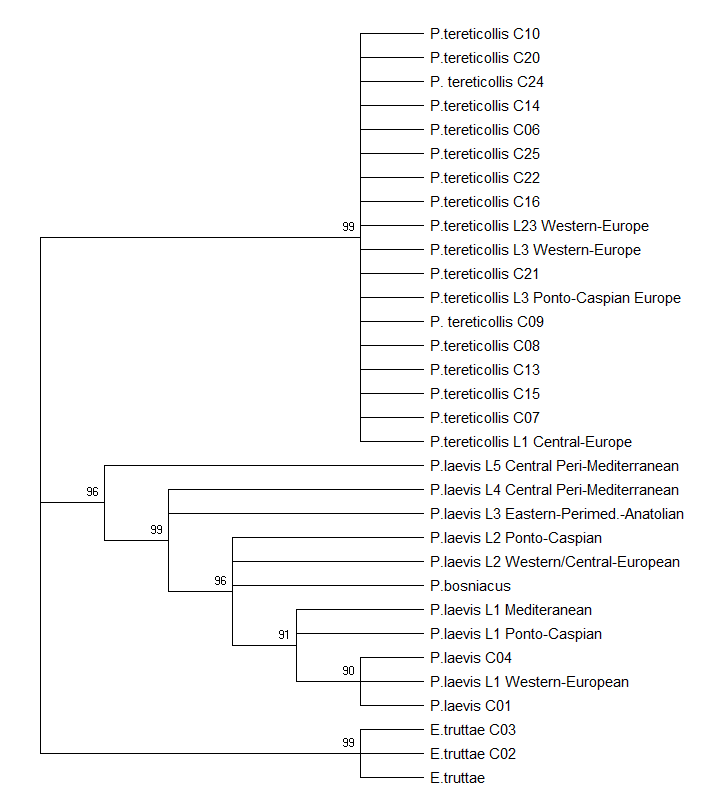


**A) Evolutionary relationships of European *Pomphorhynchidae* with *Echinorhynchus trutta as outgroup* based on the COI gene**

The evolutionary history was inferred using the Minimum Evolution method [1]. The optimal tree with the sum of branch length = 1.023 is shown. The confidence probability (multiplied by 100) that the interior branch length is greater than 0, as estimated using the bootstrap test (1000 replicates is shown next to the branches [1,2]. The tree is drawn to scale, with branch lengths in the same units as those of the evolutionary distances used to infer the phylogenetic tree. The evolutionary distances were computed using the Kimura 2-parameter method [3] and are in the units of the number of base substitutions per site. The rate variation among sites was modelled with a gamma distribution (shape parameter = 1). The ME tree was searched using the Close-Neighbour-Interchange (CNI) algorithm [4] at a search level of 1. The Neighbour-joining algorithm [5] was used to generate the initial tree. This analysis involved 32 nucleotide sequences. Codon positions included were 1st+2nd+3rd. All ambiguous positions were removed for each sequence pair (pairwise deletion option). There were a total of 614 positions in the final dataset. Evolutionary analyses were conducted in MEGA X [6].

A condensed tree is shown with a cut-off value at 90% confidence probability. Lineages *P. laevis* L1 to L5 and P. tereticollis L1 to L3 are based on consensus sequences taken from Perrot-Minot et al. [7]. *P. bosniacus* was taken from Reier et al.[8]. *Echinorhynchus truttae* was taken from NCBI accession number DQ089710.


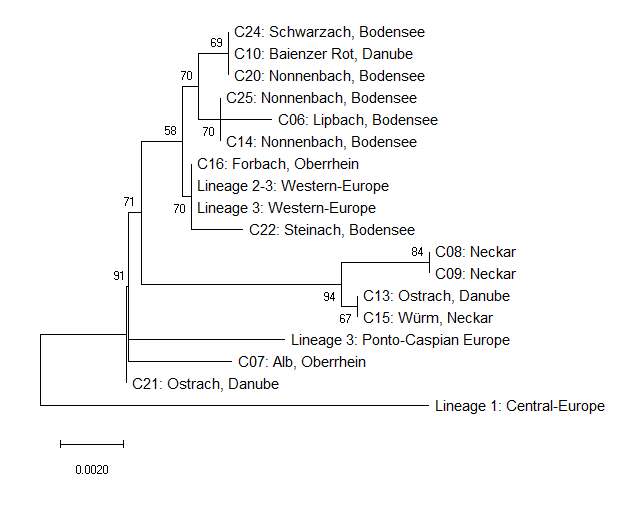


**B) Evolutionary relationships of *P. tereticollis* samples based on the COI sequence**

The evolutionary history was inferred using the Minimum Evolution method [1]. The optimal tree with the sum of branch length = 0.0406 is shown. The confidence probability (multiplied by 100) that the interior branch length is greater than 0, as estimated using the bootstrap test (1000 replicates is shown next to the branches [1,2]. The tree is drawn to scale, with branch lengths in the same units as those of the evolutionary distances used to infer the phylogenetic tree. The evolutionary distances were computed using the Kimura 2-parameter method [3] and are in the units of the number of base substitutions per site. The rate variation among sites was modelled with a gamma distribution (shape parameter = 1). The ME tree was searched using the Close-Neighbour-Interchange (CNI) algorithm [4] at a search level of 1. The Neighbour-joining algorithm [5] was used to generate the initial tree. This analysis involved 18 nucleotide sequences. Codon positions included were 1st+2nd+3rd. All ambiguous positions were removed for each sequence pair (pairwise deletion option). There were a total of 614 positions in the final dataset. Evolutionary analyses were conducted in MEGA X [6].


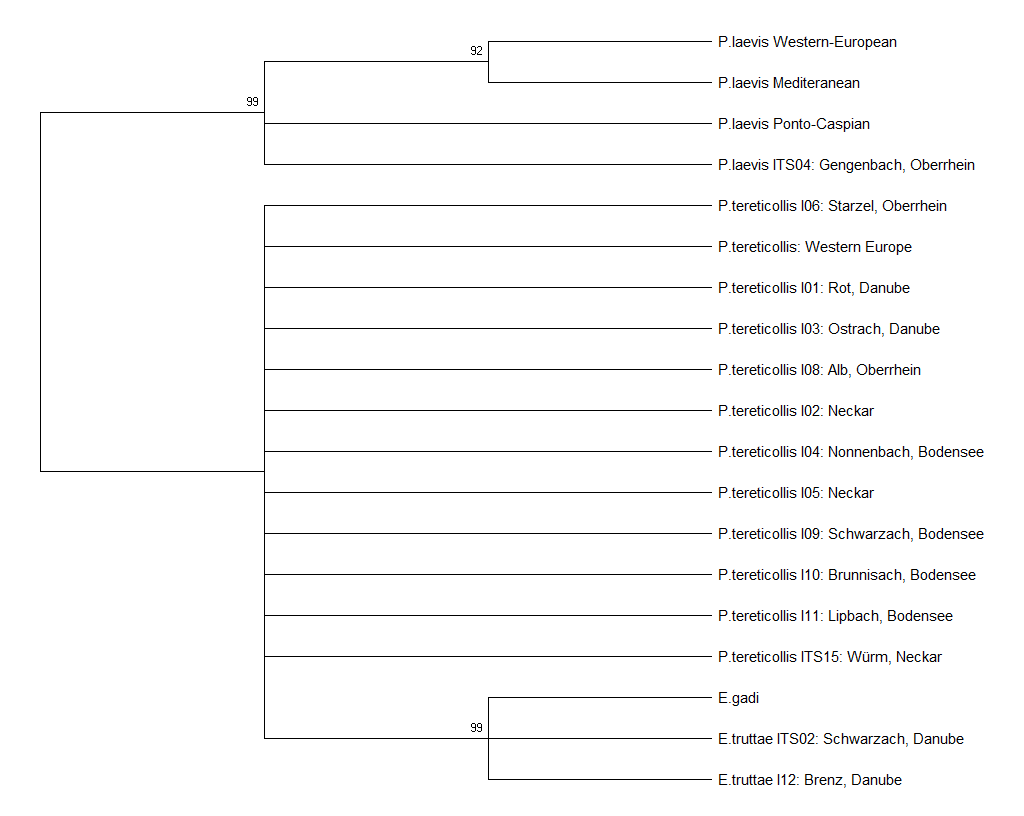


**C) Evolutionary relationships of *Echinorhynchus* and *Pomphorhynchus* samples based on the ITS sequence**

The evolutionary history was inferred using the Minimum Evolution method [1]. The optimal tree with the sum of branch length = 1.700 is shown. The confidence probability (multiplied by 100) that the interior branch length is greater than 0, as estimated using the bootstrap test (1000 replicates is shown next to the branches [1,2]. The tree is drawn to scale, with branch lengths in the same units as those of the evolutionary distances used to infer the phylogenetic tree. The evolutionary distances were computed using the Kimura 2-parameter method [3] and are in the units of the number of base substitutions per site. The rate variation among sites was modelled with a gamma distribution (shape parameter = 1). The ME tree was searched using the Close-Neighbour-Interchange (CNI) algorithm [4] at a search level of 1. The Neighbour-joining algorithm [5] was used to generate the initial tree. This analysis involved 20 nucleotide sequences. All ambiguous positions were removed for each sequence pair (pairwise deletion option). There were a total of 732 positions in the final dataset. Evolutionary analyses were conducted in MEGA X [6].

1. Rzhetsky A, Nei M. A simple method for estimating and testing minimum-evolution trees. Mol Biol Evol. 1992;9: 945–967. doi:10.1093/oxfordjournals.molbev.a040771

2. Felsenstein J. Confidence limits on phylogenies: an approach using the bootstrap. Evolution. 1985;39: 783–791.

3. Tamura K, Nei M, Kumar S. Prospects for inferring very large phylogenies by using the neighbor-joining method. Proc Natl Acad Sci. 2004;101: 11030–11035.

4. Nei M, Kumar S. Molecular evolution and phylogenetics. Oxford university press; 2000.

5. Saitou N, Nei M. The neighbor-joining method: a new method for reconstructing phylogenetic trees. Mol Biol Evol. 1987;4: 406–425. doi:10.1093/oxfordjournals.molbev.a040454

6. Kumar S, Stecher G, Li M, Knyaz C, Tamura K. MEGA X: molecular evolutionary genetics analysis across computing platforms. Mol Biol Evol. 2018;35: 1547–1549.

7. Perrot-Minnot M-J, Špakulová M, Wattier R, Kotlík P, Düşen S, Aydoğdu A, et al. Contrasting phylogeography of two Western Palaearctic fish parasites despite similar life cycles. J Biogeogr. 2018;45: 101–115. doi:10.1111/jbi.13118

8. Reier S, Sattmann H, Schwaha T, Harl J, Konecny R, Haring E. An integrative taxonomic approach to reveal the status of the genus *Pomphorhynchus* Monticelli, 1905 (Acanthocephala: Pomphorhynchidae) in Austria. Int J Parasitol Parasites Wildl. 2019;8: 145–155. doi:10.1016/j.ijppaw.2019.01.009
