# Supplementary material for: Morphological and molecular data show no evidence of the proposed replacement of endemic Pomphorhynchus tereticollis by invasive P. laevis in salmonids in southern Germany
Source: PLoS One. 2020 Jun 16;15(6):e0234116. doi: 10.1371/journal.pone.0234116 (PMC7297375; doi:10.1371/journal.pone.0234116)
Supplement: S1 Table — (DOCX) [file pone.0234116.s002.docx]

**S2 Table. Sample sites and prevalence in Brown trout (*Salmo trutta*) and grayling (*Thymallus thymallus*) in Baden-Württemberg during the survey in 2018-2019.**

|  |  | |  | |  | |  |  | |  | |  | |  |
| --- | --- | --- | --- | --- | --- | --- | --- | --- | --- | --- | --- | --- | --- | --- |
| **longitute** | **latitude** | **species** | | **drainage area** | | **river** | | | **sampled** | | **infected** | | **parasite** | |
| 8.506925 | 47.82887 | *Thymallus thymallus* | | High Rhine | | Wutach | | | 10 | | 0 | |  | |
| 8.393213 | 48.96263 |  |  | Upper Rhine | | Alb | | | 2 | | 0 | |  | |
| 8.600742 | 48.24651 |  |  | Neckar | | Neckar | | | 8 | | 3 | | *P. tereticollis* | |
| 8.854795 | 48.44961 |  |  |  |  |  |  |  | 6 | | 2 | | *P. tereticollis* | |
| 10.04238 | 48.03212 |  |  | Danube | | Baierzer Rot | | | 1 | | 0 | |  | |
| 9.895265 | 48.27723 |  |  |  |  |  |  |  | 10 | | 4 | | *P. tereticollis* | |
| 8.935632 | 48.01343 |  |  |  |  | Danube | | | 10 | | 1 | | *P. tereticollis* | |
| 8.67645 | 47.92453 |  |  |  |  |  |  |  | 3 | | 3 | | *P. tereticollis* | |
| 8.891796 | 48.0298 |  |  |  |  |  |  |  | 10 | | 3 | | *P. tereticollis* | |
| 9.474288 | 48.14099 |  |  |  |  | Schwarzach | | | 3 | | 0 | |  | |
|  |  |  | |  | | total: | | | 63 | | 16 | |  | |
|  |  |  | |  | |  | | |  | |  | |  | |
| 9.579226 | 47.61025 | *Salmo trutta* | | Lake Constance | | Argen | | | 6 | | 0 | |  | |
| 10.00168 | 47.70808 |  |  |  |  |  |  |  | 10 | | 0 | |  | |
| 9.413959 | 47.67926 |  |  |  |  | Brunnisach | | | 10 | | 6 | | *P. tereticollis* | |
| 9.731655 | 47.68485 |  |  |  |  | Haslach | | | 10 | | 0 | |  | |
| 9.764684 | 47.72506 |  |  |  |  |  |  |  | 10 | | 0 | |  | |
| 9.397571 | 47.69379 |  |  |  |  | Lipbach | | | 10 | | 7 | | *P. tereticollis* | |
| 9.591901 | 47.58989 |  |  |  |  | Nonnenbach | | | 22 | | 19 | | *P. tereticollis* | |
| 8.833277 | 47.80055 |  |  |  |  | Saubach | | | 7 | | 0 | |  | |
| 9.589943 | 47.73336 |  |  |  |  | Schwarzach | | | 10 | | 2 | | *P. tereticollis* | |
| 9.634003 | 47.70485 |  |  |  |  |  |  |  | 10 | | 5 | | *P. tereticollis* | |
| 9.752795 | 47.83860 |  |  |  |  | Wolfegger Ach | | | 10 | | 0 | |  | |
| 8.095280 | 47.63175 |  |  | High Rhine | | Hauensteiner Alb | | | 12 | | 2 | | *P. tereticollis* | |
| 8.129230 | 47.59041 |  |  |  |  |  |  |  | 4 | | 1 | | *P. tereticollis* | |
| 8.142801 | 47.73393 |  |  |  |  |  |  |  | 21 | | 0 | |  | |
| 8.250521 | 47.65767 |  |  |  |  | Schlücht | | | 10 | | 3 | | *P. tereticollis* | |
| 8.346557 | 47.73922 |  |  |  |  | Steina | | | 6 | | 0 | |  | |
| 7.908730 | 47.63753 |  |  |  |  | Wehra | | | 12 | | 7 | | *P. tereticollis* | |
| 7.752678 | 47.64374 |  |  |  |  | Wiese | | | 9 | | 0 | |  | |
| 7.888714 | 47.77244 |  |  |  |  |  |  |  | 10 | | 0 | |  | |
| 7.954209 | 47.83267 |  |  |  |  |  |  |  | 12 | | 0 | |  | |
| 8.251803 | 47.87967 |  |  |  |  | Wutach | | | 13 | | 0 | |  | |
| 8.346619 | 47.84421 |  |  |  |  |  |  |  | 2 | | 0 | |  | |
| 8.351852 | 47.64585 |  |  |  |  |  |  |  | 10 | | 0 | |  | |
| 8.380737 | 47.67703 |  |  |  |  |  |  |  | 10 | | 0 | |  | |
| 8.398546 | 47.84277 |  |  |  |  |  |  |  | 2 | | 0 | |  | |
| 8.449850 | 47.84848 |  |  |  |  |  |  |  | 1 | | 0 | |  | |
| 8.494285 | 47.78200 |  |  |  |  |  |  |  | 2 | | 0 | |  | |
| 8.506925 | 47.82887 |  |  |  |  |  |  |  | 10 | | 0 | |  | |
| 8.393213 | 48.96263 |  |  | Upper Rhine | | Alb | | | 10 | | 4 | | *P. tereticollis* | |
| 8.198530 | 48.65635 |  |  |  |  | Bühlot | | | 10 | | 6 | | *P. tereticollis* | |
| 7.862060 | 47.99079 |  |  |  |  | Dreisam | | | 12 | | 0 | |  | |
| 7.891418 | 47.99062 |  |  |  |  |  |  |  | 9 | | 1 | | *P. tereticollis* | |
| 8.094453 | 48.35667 |  |  |  |  | Erlenbach | | | 10 | | 0 | |  | |
| 8.376031 | 48.48292 |  |  |  |  | Forbach | | | 9 | | 7 | | *P. tereticollis* | |
| 7.968522 | 48.43509 |  |  |  |  | Kinzig | | | 1 | | 0 | |  | |
| 8.013178 | 48.40104 |  |  |  |  |  |  |  | 1 | | 0 | |  | |
| 8.111360 | 48.28175 |  |  |  |  |  |  |  | 1 | | 0 | |  | |
| 8.201755 | 48.26861 |  |  |  |  |  |  |  | 18 | | 0 | |  | |
| 8.388500 | 48.31871 |  |  |  |  |  |  |  | 11 | | 0 | |  | |
| 8.346948 | 48.74107 |  |  |  |  | Murg | | | 10 | | 0 | |  | |
| 8.146962 | 48.48858 |  |  |  |  | Rench | | | 10 | | 0 | |  | |
| 7.945717 | 48.29691 |  |  |  |  | Schutter | | | 10 | | 9 | | *P. tereticollis* | |
| 8.952769 | 48.36596 |  |  |  |  | Starzel | | | 5 | | 4 | | *P. tereticollis* | |
| 9.133671 | 49.34904 |  |  | Neckar | | Elz | | | 6 | | 0 | |  | |
| 9.198284 | 49.40208 |  |  |  |  |  |  |  | 8 | | 0 | |  | |
| 9.420770 | 48.46830 |  |  |  |  | Erms | | | 10 | | 1 | | *P. tereticollis* | |
| 8.766870 | 48.43079 |  |  |  |  | Eyach | | | 5 | | 0 | |  | |
| 8.832587 | 48.34121 |  |  |  |  |  |  |  | 2 | | 0 | |  | |
| 9.197665 | 48.70425 |  |  |  |  | Körsch | | | 5 | | 2 | | *P. tereticollis* | |
| 8.732582 | 48.73089 |  |  |  |  | Nagold | | | 5 | | 0 | |  | |
| 8.600742 | 48.24651 |  |  |  |  | Neckar | | | 6 | | 0 | |  | |
| 8.854795 | 48.44961 |  |  |  |  |  |  |  | 1 | | 0 | |  | |
| 9.849061 | 48.80754 |  |  |  |  | Rems | | | 10 | | 0 | |  | |
| 9.237813 | 49.28510 |  |  |  |  | Schefflenz | | | 2 | | 0 | |  | |
| 9.257961 | 49.35190 |  |  |  |  |  |  |  | 10 | | 0 | |  | |
| 8.858422 | 48.46421 |  |  |  |  | Seltenbach | | | 10 | | 0 | |  | |
| 8.860005 | 48.43612 |  |  |  |  | Starzel | | | 4 | | 0 | |  | |
| 8.880757 | 48.38652 |  |  |  |  |  |  |  | 2 | | 1 | | *P. tereticollis* | |
| 9.005431 | 48.34907 |  |  |  |  |  |  |  | 8 | | 0 | |  | |
| 8.801538 | 49.46675 |  |  |  |  | Steinach | | | 10 | | 6 | | *P. tereticollis* | |
| 8.835571 | 48.78684 |  |  |  |  | Würm | | | 10 | | 2 | | *P. tereticollis* | |
| 9.995657 | 48.11921 |  |  | Danube | | Baierzer Rot | | | 3 | | 0 | |  | |
| 10.04238 | 48.03212 |  |  |  |  |  |  |  | 1 | | 0 | |  | |
| 8.899432 | 48.10389 |  |  |  |  | Bära | | | 8 | | 0 | |  | |
| 9.964950 | 48.39859 |  |  |  |  | Blau | | | 10 | | 0 | |  | |
| 10.15470 | 48.62980 |  |  |  |  | Brenz | | | 10 | | 0 | |  | |
| 10.28070 | 48.57240 |  |  |  |  |  |  |  | 7 | | 2 | | *E. trutta* | |
| 9.552256 | 48.08825 |  |  |  |  | Kanzach | | | 6 | | 1 | | *P. laevis* | |
| 9.288988 | 48.10417 |  |  |  |  | Lauchert | | | 10 | | 0 | |  | |
| 10.09435 | 47.83850 |  |  |  |  | Lautracher Ach | | | 11 | | 0 | |  | |
| 9.368648 | 48.03635 |  |  |  |  | Ostrach | | | 10 | | 9 | | *P. tereticollis* | |
| 9.475010 | 48.04211 |  |  |  |  | Schwarzach | | | 10 | | 2 | | *E. trutta* | |
|  |  |  | |  | | total: | | | 588 | | 109 | |  | |
|  |  |  | |  | |  | | |  | |  | |  | |
