# Supplementary material for: Morphological and molecular data show no evidence of the proposed replacement of endemic Pomphorhynchus tereticollis by invasive P. laevis in salmonids in southern Germany
Source: PLoS One. 2020 Jun 16;15(6):e0234116. doi: 10.1371/journal.pone.0234116 (PMC7297375; doi:10.1371/journal.pone.0234116)
Supplement: S2 Table — (DOCX) [file pone.0234116.s003.docx]

**S3 Table. Examples of Sanger sequences of acanthocephalans in Baden-Württemberg.**

3.1) *P. laevis*Partial sequences of the Cytochrome c oxidase subunit 1 (COI) and internal transcribed spacers 1 (ITS) DNA. The specimens found in this chub captured in the Kinzig were used for reverence in comparison with the specimens found in the salmonids.

3.2) *P. tereticollis*Partial sequences of the Cytochrome c oxidase subunit 1 (COI) and internal transcribed spacers (ITS) DNA. Obtained from a brown trout.

3.2) *P. tereticollis*Partial sequences of the Cytochrome c oxidase subunit 1 (COI) and internal transcribed spacers (ITS) DNA. Obtained from a brown trout.

3.3) *E. trutta*Partial sequences of the Cytochrome c oxidase subunit 1 (COI) and internal transcribed spacers (ITS) DNA. Obtained from a brown trout.

3.4) *P. tereticollis*Partial sequences of the Cytochrome c oxidase subunit 1 (COI) and inner sequence of COI gene. Obtained from a brown trout.

| 3.1 |
| --- |
| Species: *Pomphorhynchus laevis*  Locality: Kinzig bei Gengenbach Host: *Leuciscus cephalus* |
| forward primer: LCO1490 reverse primer: HCO2198 Code: BW004-20 COI sequence (5'->3') 612 bases |
| TTTGATGTATGTTTTGGTTGGTGTGTGAGGGGGGCTAATGGGGTTTTCTATAAGACTATTAATTCGATTAGAATTAGGGAGAGGAGGGGTTTGGATAGGAAGAGAGGCTGTGTATAATGTTTTAGTAACCAGACACGCTGTTATAATGGTATTTTTTCTAGTAATACCAGTATTTATGGGAGGGTTCGGTAATTGACTCATGCCAGTGATGTTAGGGTTGAGGGATATGGCCCTCCCCCGACTGAACAATTTGAGGCTTATCCTACTTCTTGCTAGGTTGGGGGTTATAGGTGTGTCCTTGCTTTTAGGAGGGGGTGGGGCTGGTTGAACAATGTACCCACCCCTAATGTTAGGGGATTATAGGTCCGGTGTAGCTGTTGATCTAATGATTTTGAGGTTGCATGTAGTAGGTCTTTCCTCTATTCTAGGTTCAATCAATATCTTGATTACCTGGGTAGCCGGGAGAAGGGTGGTGTATAGAGTAGAACAGACGCCCCTATTCGTGTGGGCTTTAGTAACGACTGCGGGTCTAGTAGTTTTAACAGTCCCAGTCTTGGCGGCAGCTTTAACCATACTTTTGATAGATCGTAATTTGAACACCAGATTTTTCGA |
| forward primer: ITS_BD1 reverse primer: ITS_BD2 Code: ITS04 ITS sequence (5'->3') 662 bases |
| GGTGAACCTGCAGAAGGATCATTGACGCTGAAACCACCTGTATAGGTGGGAACAATCTTGACTGTTACTTCTGTTGTTGACTGATTGATCATGATGAATTCCACGTAAGGGTAAGGTGGAGTTCTAATTATTATTGTCATGTGAATATGACTTGCCTCGATTATAAACTGTTGTTTACTGAAACTGGATTTGTGGCAATAATATAATGTTCTTGAGCTAGGCAAGAACTATCATTTACTTGTTGATGGTAGCTAAAACAAGAAATGAACGTCGGTGTACAGTGAATCACTTGGCTCGCAAATCGAGGAAGAACGTTGCCATATGTGATAAATTCTGTGAACTGCAGGACACACGGAACAGATAGTTTTTGAACGCAAATGACAGCTGTGAGGTCAAACTCATAGCTACATCCGTTTGAGGTCGATTAATATGAATATATTAACAGTGTTAGTGATGACTACATAATAAATAACGCTGCTAACGATAAGTGCATAATGCAGGCGAGCAATTCTAATCAGTGACTATATATAGAAGAACATGCTAGCTGAACTGCACTATATCTTGAAACACTGACGATAACTCTGCGTAACTGGGTATTGATAATCATATGAGTGAAGTGTCGTCGTGATTGATCGATATTTGGCCTCAAATCGGGTGAGATA |

| 3.2 |
| --- |
| Species: *Pomphorhynchus tereticollis*  Locality: Würm bei Hausen alte Bogenbrücke Host: *Salmo trutta* |
| forward primer: LCO1490  reverse primer: HCO2198 Code: COI15 COI sequence (5'->3') 612 bases |
| TTTAATATATATTTTGGTTGGTGTTTGGGGAGGATTGATGGGGTTTTCTATAAGGCTACTAATTCGATTAGAGTTAGGGAGAGGGGGAGTTTGGATGGGTAGAGAGGCCATCTATAACGTATTGGTGACTAGACATGCAGTTATGATAGTATTCTTTTTAGTTATACCAGTTTTTATGGGGGGGTTTGGTAATTGACTGATACCTGTTATATTAGGGTTGAGGGACATGGCCCTACCCCGTTTAAATAATTTGAGGTTGATTTTACTACTTGCTAGGTTAGGGATTATGGGGGTATCATTACTGTTGGGTGGAGGTGGAGCCGGTTGAACAATGTACCCACCCCTCATGTTGAGGGATTACAGGTCTGGGGTAGCTGTTGACTTAATAATTTTGGGGTTGCATGTAGTGGGGTTGTCATCGATTTTAGGTTCAATTAACATTTTGGTTACTTGGGTGGCAGGGAGGAGCGTAGTATATAGTGTAGAGCAAGCCCCGTTATTTGTATGGGCTATAGTGACAACTGCGGGATTAGTGGTTTTGACGGTACCGGTTTTGGCAGCAGCATTAACGATACTTTTGATGGACCGTAATTTGAACTCCAGGTTTTTTGA |
| forward primer: ITS_BD1 reverse primer: ITS_BD2 Code: ITS15 ITS sequence (5'->3') 690 bases |
| GGTGAACCTGCAGAAGGATCATTGACGCTGAAACCACCTGTATAGGTGGGAACAATCTTGACTGTTACTTCTATTGTTGACTGATTGATCATGATGAATTCCACGTAAGGGTAAGGTGGAGTTCTAATTGTTGTTGTCATGTGAATATGACTTGCCTCGTTTATAAACTGTTATTTACAGAACTGAAACTGGTTTTGTGGCAATATACTGGTTTGTGGCAATAATATCTGAATGTTCTTGAGCTAGGCAAGAACTATCATTTACTTGTTGATGGTAGCTAAAACAAGAAATGAACGTCGGTGTACAGTGAATCACTTGGCTCGCAAATCGAGGAAGAACGTTGCCATATGTGATAAATTCTGTGAACTGCAGGACACACGGAACAGATAGTTTTTGAACGCAAATGACAGCTGTGAGGTCAAACTCATAGCTACATCCGTTTGAGGTCGATAAATATATGAATATATTAACAGTGTTAATGATGATTGCATACTAAACAACGCTGTCAACGATAAGTGCAAAATGCAGGCTAGCCATTCTAATCAGTGACTATATATGTAGAATATGCTTGCAGAACCTGCACTATATCTTGAAAAACTGACGATAACTCTGCGTGCCTGGGAATTGATAATCATATGAGTGAATTATCGTCGTGATTGATCGATATTTGGCCTCAAATCGGGTGAGATA |

| 3.3 |
| --- |
| Species: *Echinorhynchus truttae*  Locality: Danube, Locality: Schwarzach bei Lindenmühle  Host: *Salmo trutta* |
| forward primer: LCO1490 reverse primer: HCO2198  Code: COI02  COI sequence (5'->3') 612 bases |
| GTTAATATATTTTTTAGTGAGGATTTGGGGTGGTTTAGTGGGTTTTAGTTTAAGCTTGTTAATTCGGTTAGAGCTGGGTTCTGGAGGGCAGTGAATAGGTGATGAGCATTTGTATAATGTAGTGGTTACTGCACATGCTATTATAATAGTGTTCTTTTTAGTTATACCTATGTTTATGGGGGGTTTTGGTAATTGGTTAATGCCCGTTATATTGGGGTTAAGAGATATGGTGTTACCTCGTTTAAATAATTTAAGTTTTTTAATTTTACCTTTTAGTTTGTTGCTTATGGCTTCGTCTTTGATGCTGAAAGGCGGTGGGGCCGGTTGGACAATGTACCCACCACTAATATTAAGAGATTATAGTTCTGGTGTTTCAGTTGATATAATAATTTTGAGTTTACATTTAGCTGGGTTATCTTCTATTTTGGGTTCTATTAATGTAATAGTAACAGGAGTTGTAGGATCTAAAATTGCTGGTAGTGTTGAGCAGTTGCCTCTGCTTATTTGAGCACTGTTAGTTACAGCTGGGCTTGTGCTTTTGACGGTTCCTGTTTTAGCTGCAGCTTTAACAATGCTTTTGTTAGACCGTAACTTTAGGTCTAGATTTTTTGA |
| forward primer: ITS_BD1 reverse primer: ITS_BD2  Code: ITS02  ITS sequence (5'->3') 576 bases |
| GGTGAACCTGCGGAAGGATCATTGAAGTCTTGACCATCATTTTTGTGATGGATACGGCTATAAAGAAATTCTGTGAAAAATTGGCAACCCCATGTACGGGTAAGGTGGATTGCTGATACAAAACTTAAATGACTTACCTGTCCAGTAATTAGGGTATGATTAATTGATAATGTTTGCGACTTGCAGACTATATTACATACTTTTTTGGTGAAAACTCAATTCTTGAAATGAATTGTCAGTGTACAGTGAATCACTTGGCTCGCAAAACGAGGAAGGACATTGCCATATGTGATAAATTCTGTGAACTGCAGATCACACGGAACAGACAGTTTTTGAACGCAAATGACAGTTGTGAGTTTTTGCTCACAACTACACCCGTTTGAGACCTTAAAAATTTATAACTCAATATAGTTGTAAACAGTTGATGTATGATTAATGTTGGTGTATTCTGTTAATTGAAATGACCATTATGTGTGTCAAACTGAGATTATCTAACACTGCCTTGAAATGCTCTGTGGAATGAGAATAATTGATGGTGGTTGTTAACTTATGGTCTAAATCATTTGGTCTCAAATC |

| 3.4 |
| --- |
| Species: *Pomphorhynchus tereticollis*  Locality: Nonnenbach, Kressbronn Host: *Salmo trutta* |
| forward primer: T2-fw reverse primer: T1-rev Code: PTN COI inner sequence (5'->3') 216 bases positions 365-580 COI sequence |
| TCTGGGGTAGCTGTTGACTTAATAATTTTGGGGTTGCATGTAGTGGGGTTGTCATCGATTTTAGGTTCAATTAATATTTTGGTTACTTGGGTGGCAGGGAGGAGCGTAGTATATAGTGTAGAGCAAGCCCCGTTATTTGTATGGGCTATAGTGACAACTGCGGGATTAGTGGTTTTGACGGTACCGGTTTTGGCAGCAGCATTAACGATACTTTTG |
| forward primer: HCO2198 reverse primer: LCO1490 Code: COI14 COI sequence (5'->3') 655 bases |
| TTTAATATATATTTTGGTTGGTGTGTGGGGAGGATTGATGGGGTTTTCTATAAGGCTACTAATTCGATTAGAGTTAGGGAGAGGGGGAGTTTGGATGGGTAGAGAGGCCATCTATAACGTATTGGTGACTAGGCATGCAGTTATGATAGTATTCTTTTTAGTTATACCAGTTTTTATGGGGGGGTTTGGTAACTGACTAATACCTGTTATATTAGGGTTGAGGGACATGGCCCTACCCCGTTTAAATAATTTGAGGTTGATTTTACTACTTGCTAGGTTAGGGATTATGGGGGTATCATTACTGTTGGGTGGAGGTGGGGCCGGTTGAACAATGTACCCACCCCTCATGTTGAGGGATTACAGGTCTGGGGTAGCTGTTGACTTAATAATTTTGGGGTTGCATGTAGTGGGGTTGTCATCGATTTTAGGTTCAATTAATATTTTGGTTACTTGGGTGGCAGGGAGGAGCGTAGTATATAGTGTAGAGCAAGCCCCGTTATTTGTATGGGCTATAGTGACAACTGCGGGATTAGTGGTTTTGACGGTACCGGTTTTGGCAGCAGCATTAACGATACTTTTGATGGACCGTAATTTGAACTCTAGGTTTTTTGACCCTTGTGGAGGGGGTTCGCCAATTCTGTATCAACACTTATTT |
