# Supplementary material for: Morphological and molecular data show no evidence of the proposed replacement of endemic Pomphorhynchus tereticollis by invasive P. laevis in salmonids in southern Germany
Source: PLoS One. 2020 Jun 16;15(6):e0234116. doi: 10.1371/journal.pone.0234116 (PMC7297375; doi:10.1371/journal.pone.0234116)
Supplement: S3 Table — (DOCX) [file pone.0234116.s004.docx]

**S4 Table. Consensus sequences used for genetic analyses.**

>E.truttae

ggGTTGATATATTTTTTAGTGAGGATTTGAGGTGGTTTAGTGGGTTTTAGTTTAAGCTTGTTAATTCGGTTAGAGCTGGG

TTCTGGAGGGCAGTGAATAGGTGATGAGCATTTGTATAATGTAGTGGTTACTGCACATGCTATTATAATAGTGTTCTTTT

TAGTTATACCTATGTTTATGGGGGGTTTTGGTAATTGGTTAATGCCCGTTATATTGGGGTTAAGAGATATGGTGTTACCT

CGTTTAAATAATTTAAGTTTTTTAATTTTACCTTTTAGTTTGTTGCTTATGGCTTCGTCTTTGATGCTGAAAGGCGGTGG

GGCCGGTTGGACAATGTACCCACCACTAATATTAAGAGATTATAGTTCTGGTGTTTCAGTTGATATAATAATTTTGAGTT

TACATTTAGCTGGGTTATCTTCTATTTTGGGTTCTATTAATGTAATAGTAACAGGAGTTGTAGGATCTAAAATTGCTGGT

AGTGTTGAGCAGTTGCCTCTGCTTATTTGAGCACTGTTAGTTACAGCTGGGCTTGTGCTTTTGACGGTTCCTGTTTTAGC

TGCAGCTTTAACAATGCTTTTGTTAGACCGTAACTTTAGGTCTAGATTTTTTGA

>P.bosniacus

ggtTTGATGTATGTTTTGGTTGGTGTGTGAGGGGGGCTAATGGGGTTTTCTATAAGGTTATTAATTCGATTAGAGTTGGG

GAGAGGAGGGGTTTGGATAGGGAGGGAGGCTGTGTATAATGTGTTAGTAACTAGACACGCTGTTATAATGGTCTTTTTTC

TAGTAATACCGGTATTTATGGGGGGATTCGGTAATTGGCTCATGCCAGTGATGTTGGGGTTAAGGGATATGGCCCTTCCC

CGATTGAACAATTTAAGGCTTATCCTGCTTCTCGCTAGGCTGGGTATTATAGGAGTATCTTTGCTTTTAGGAGGGGGTGG

TGCTGGTTGGACAATATATCCCCCACTGATGTTAGGGGACTATAGGTCTGGTGTAGCTGTTGATCTAATGATCCTGAGGT

TGCATGTAGTAGGTCTTTCTTCTATTCTAGGCTCAATCAATATCCTGATTACCTGGGTAGCTGGAAGAAGGGTAGTGTAT

AGAGTAGAACAGGCGCCACTGTTCGTATGGGCTTTAGTAACGACGGCTGGTCTAGTAGTTTTAACGGTCCCAGTCTTGGC

GGCAGCCTTGACCATACTTTTAATGGATCGTAATTTGAATTCCAGGTTTTTTGA

>P.laevis_L1_Mediteranean

GGTCTGATGTATGTTTTGGTTGGTGTGTGAGGGGGGCTAATGGGATTTTCTATAAGACTACTAATTCGATTAGAATTAGG

GAGAGGAGGGGTTTGGATAGGAAGAGAGGCTGTGTATAATGTTTTAGTAACCAGACACGCTGTTATAATGGTATTTTTTC

TAGTAATACCAGTATTTATGGGAGGATTCGGTAATTGACTCATGCCAGTGATGTTAGGGTTGAGGGATATAGCCCTCCCC

CGGCTGAACAATTTGAGGCTTATCTTACTTCTCGCTAGGTTGGGGGTTATAGGTGTGTCTTTGCTTTTAGGAGGGGGTGG

GGCCGGTTGGACAATGTACCCACCCCTAATGTTAGGGGATTATAGGTCCGGTGTAGCTGTTGATCTAATGATTTTGAGGT

TACATGTAGTAGGTCTTTCCTCTATTCTAGGTTCAATCAATATCTTGATTACCTGGGTAGCCGGGAGAAGGGTGGTGTAT

AGAGTAGAACAGACACCACTGTTCGTGTGGGCTTTAGTGACGACTGCGGGTCTAGTAGTTTTAACAGTCCCAGTCTTGGC

GGCAGCCTTAACTATGCTTTTGATGGACCGTAATTTGAATCCCAGATTTTTCGA

>P.laevis_L1_Ponto-Caspian

GGTCTGATGTATGTTTTGGTTGGTGTGTGAGGGGGGCTAATGGGGTTTTCTATAAGACTACTAATTCGATTAGAATTAGG

GAGAGGAGGGGTTTGGATGGGAAGAGAGGCTGTGTATAATGTTTTAGTAACCAGACACGCTGTTATAATGGTATTTTTTC

TAGTAATACCAGTATTTATGGGAGGATTCGGTAATTGACTCATGCCAGTGATGTTAGGGTTGAGGGATATAGCCCTCCCC

CGGCTGAACAATTTGAGGCTTATCTTACTTCTCGCTAGGTTGGGGGTTATAGGTGTGTCCTTGCTTTTAGGAGGGGGTGG

GGCCGGTTGGACAATGTACCCACCCCTAATGTTAGGGGATTATAGGTCCGGTGTAGCTGTTGATCTAATGATTTTGAGGT

TGCATGTAGTAGGTCTTTCCTCTATTCTAGGTTCAATCAATATCTTGATTACCTGGGTAGCCGGGAGAAGAGTGGTGTAT

AGAGTAGAACAGACACCACTGTTCGTGTGGGCTTTAGTAACGACTGCGGGTCTAGTAGTTTTAACAGTCCCAGTCTTGGC

GGCAGCCTTAACCATGCTTTTGATGGACCGTAATTTGAATACCAGATTTTTCGA

>P.laevis_L1_Western-European

GGTTTGATGTATGTTTTGGTTGGTGTGTGAGGGGGGCTAATGGGGTTTTCTATAAGACTATTAATTCGATTAGAATTAGG

GAGAGGAGGGGTTTGGATAGGAAGAGAGGCTGTGTATAATGTTTTAGTAACCAGACACGCTGTTATAATGGTATTTTTTC

TAGTAATACCAGTATTTATGGGAGGGTTCGGTAATTGACTCATGCCAGTGATGTTAGGGTTGAGGGATATGGCCCTCCCC

CGACTGAACAATTTGAGGCTTATCCTACTTCTTGCTAGGTTGGGGGTTATAGGTGTGTCCTTGCTTTTAGGAGGGGGTGG

GGCTGGTTGAACAATGTACCCACCCCTAATGTTAGGGGATTATAGGTCCGGTGTAGCTGTTGATCTAATGATTTTGAGGT

TGCATGTAGTAGGTCTTTCCTCTATTCTAGGTTCAATCAATATCTTGATTACCTGGGTAGCCGGGAGAAGGGTGGTGTAT

AGAGTAGAACAGACGCCTCTATTCGTGTGGGCTTTAGTAACGACTGCGGGTCTAGTAGTTTTAACAGTCCCAGTTTTGGC

GGCAGCTTTAACCATGCTTTTGATGGATCGTAATTTGAACACCAGATTTTTCGA

>P.laevis_L2_Ponto-Caspian

GGTTTGATGTATGTTTTGGTTGGTGTGTGAGGGGGGCTAATGGGGTTTTCTATAAGGTTATTAATTCGATTAGAGTTGGG

GAGAGGAGGGGTTTGGATAGGGAGGGAGGCTGTGTATAATGTGTTAGTAACTAGACACGCTGTTATAATGGTCTTTTTTC

TAGTAATACCGGTATTTATGGGGGGATTCGGTAATTGGCTCATGCCAGTGATGTTGGGGTTAAGGGATATGGCCCTTCCC

CGATTGAACAATTTAAGGCTTATCCTGCTTCTCGCTAGGCTGGGTATTATAGGAGTATCTTTGCTTTTAGGAGGGGGTGG

TGCTGGTTGGACAATATATCCCCCACTGATGTTAGGGGACTATAGGTCTGGTGTAGCTGTTGATCTAATGATCCTGAGGT

TGCATGTAGTAGGTCTTTCTTCTATTCTAGGCTCAATCAATATCCTGATTACCTGGGTAGCTGGAAGAAGGGTAGTGTAT

AGAGTAGAACAGGCGCCACTGTTCGTATGGGCTTTAGTAACGACGGCTGGTCTAGTAGTTTTAACGGTCCCAGTCTTGGC

GGCAGCCTTGACCATACTTTTAATGGATCGTAATTTGAATTCCAGGTTTTTTGA

>P.laevis_L2_Western/Central-European

GGTTTGATGTATGTTTTGGTTGGTGTGTGAGGGGGGCTAATGGGGTTTTCTATAAGGTTATTAATTCGATTAGAGTTGGG

GAGAGGAGGGGTTTGGATAGGGAGGGAGGCTGTGTATAATGTGTTAGTAACTAGACACGCTGTTATAATGGTCTTTTTTC

TAGTAATACCGGTATTTATGGGGGGATTCGGTAATTGGCTCATGCCAGTGATGTTGGGGTTAAGGGATATGGCCCTTCCC

CGATTGAACAATTTAAGGCTTATCCTGCTTCTCGCTAGGCTGGGTATTATAGGAGTATCTTTGCTTTTAGGAGGGGGTGG

TGCTGGTTGGACAATATATCCCCCACTGATGTTAGGGGACTATAGGTCTGGTGTAGCTGTTGATCTAATGATCCTGAGGT

TGCATGTAGTAGGTCTTTCTTCTATTCTAGGCTCAATCAATATCCTGATTACCTGGGTAGCTGGAAGAAGGGTAGTGTAT

AGAGTAGAACAGGCGCCACTGTTCGTATGGGCTTTAGTAACGACGGCTGGTCTAGTAGTTTTAACGGTCCCAGTCTTGGC

GGCAGCCTTGACCATACTTTTAATGGATCGTAATTTGAATTCCAGGTTTTTTGA

>P.laevis_L3_Eastern-Perimed.-Anatolian

GGTCTGATGTATGTTTTGGTTGGTGTGTGAGGGGGGCTAATGGGATTTTCTATAAGGCTATTAATTCGATTAGAGCTAGG

GAGAGGAGGGGTTTGGATAGGAAGAGAGGCTGTGTATAATGTTTTAGTGACTAGACATGCTGTTATAATAGTATTTTTTC

TAGTAATACCAGTATTTATGGGAGGATTTGGTAATTGGCTCATGCCAGTTATGTTAGGATTGAGGGACATGGCCCTCCCA

CGACTGAATAATTTGAGGCTTATTCTACTTATCGCTAGGTTGGGAATTATAGGAGTATCCCTGCTTTTAGGAGGGGGTGG

GGCTGGTTGGACAATGTAyCCACCCCTCATGTTGGGGGATTACAGGTCTGGTGTAGCTGTTGACCTAATGATCCTGAGGT

TGCATGTAGTAGGTCTTTCCTCTATCCTAGGCTCAATCAACATCCTGATTACATGGGTAGCCGGGAGGAGGGTGGTGTAT

AGAGTAGAACAGGCACCTCTGTTTGTATGGGCTTTAGTAACGACCGCTGGCTTAGTGGTTTTAACGGT?CCAGTCTTGGC

GGCAGCTTTAACGATGCTTTTGATAGACCGTAATTTGAATGCCAGATTTTTTGA

>P.laevis_L4_Central_Peri-Mediterranean

GGTCTGATATATGTATTGGTTGGTGTGTGAGGGGGGTTAATGGGGTTTTCTATAAGGCTATTAATTCGATTAGAGTTAGG

GAGAGGAGGGGTTTGGATAGGTAGAGAGGCTGTGTATAATGTTTTAGTAACTAGACACGCCGTTATAATGGTATTTTTTC

TAGTAATACCAGTATTTATAGGGGGATTCGGTAACTGGCTCATGCCAGTGATGTTAGGGTTGAGGGATATGGCTCTACCA

CGCCTAAATAATTTGAGGCTTATTCTCCTTCTTGCCAGGTTGGGAATTATAGGTGTGTCCTTGCTTTTAGGAGGGGGTGG

GGCTGGTTGGACAATGTACCCCCCTCTTATATTAGGGGATTACAGATCGGGGGTAGCTGTTGACCTGATAATTTTGAGGC

TACATGTAGTAGGTCTTTCCTCCATTTTAGGTTCAATTAACATCTTAATTACCTGAGTAGCCGGGAGAAGGGTGGTGTAT

AGGGTAGAACAGGCACCCCTGTTTGTATGGGCTTTAGTAACGACTGCCGGCCTAGTAGTTTTAACTGTCCCAGTTTTAGC

GGCAGCTTTAACTATGCTTTTGATAGATCGTAATTTGAATGCCAGGTTTTTTGA

>P.laevis_L5_Central_Peri-Mediterranean

GGGTTGATGTATATTTTGGTTGGTGTATGAGGGGGATTATTAGGATTTTCTATAAGGTTATTAATTCGGTTAGAGCTAGG

AAGAGGGGGAGTTTGAATGGGTAGAGAGGCTGTATATAATGTATTAGTAACTAGACACGCTGTTATAATAGTATTCTTTC

TAGTTATACCTGTGTTTATGGGGGGGTTTGGTAATTGACTCATGCCGGTGATGCTTGGGTTGAGAGATATAGCTTTACCG

CGGCTAAATAATTTAAGGCTTATTTTACTTCTTGCCAGATTGGGGATTATAGGAGTATCTCTGTTATTGGGAGGGGGTGG

GGCTGGTTGGACAATATACCCACCCCTGATATTGGGAGATTATAGATCTGGGGTAGCTGTTGATATGATGATCTTGAGGT

TACATGTAGTAGGTCTTTCCTCTATTTTAGGGTCAATTAATATTTTAGTTACTTGGGTAGCTGGAAGAAGAGTAATTTAT

AGGGTAGAGCAGACACCGCTATTTGTGTGAGCCTTAGTAACGACTGCGGGTTTAGTAGTTTTAACGGTTCCAGTTTTGGC

GGCTGCTTTAACGATACTTTTGATAGACCGTAATTTAAATGCCAGGTTTTTTGA

>P.tereticollis_L1_Central-Europe

GGTTTAATATATATTTTGGTTGGTGTGTGGGGAGGATTGATGGGGTTTTCTATAAGGCTACTAATTCGATTAGAGTTAGG

GAGAGGGGGAGTTTGGATGGGTAGAGAGGCCATCTATAACGTATTGGTGACTAGACATGCAGTTATGATAGTATTCTTTT

TAGTTATACCAGTTTTTATGGGGGGGTTTGGTAACTGACTAATACCTGTTATATTAGGGTTGAGGGACATGGCCCTACCT

CGCTTAAATAATTTGAGGTTGATTTTACTACTTGCTAGGTTAGGTATTATGGGGGTATCATTACTATTGGGTGGAGGTGG

GGCCGGTTGAACAATGTACCCTCCCCTCATGTTGAGGGATTACAGGTCTGGGGTGGCTGTTGACTTAATAATTTTGGGGT

TGCATGTAGTGGGGTTGTCATCTATTTTAGGTTCAATTAATATTTTGGTTACTTGGGTGGCAGGGAGGAGCGTAGTATAT

AGTGTAGAGCAAGCCCCGTTATTTGTATGGGCTATGGTGACAACTGCGGGATTAGTGGTTTTGACGGTACCGGTTTTGGC

AGCAGCATTAACGATGCTTTTGATGGACCGTAATTTGAACTCCAGGTTTTTTGA

>P.tereticollis_L23_Western-Europe

GGTTTAATATATATTTTGGTTGGTGTGTGGGGAGGATTGATGGGGTTTTCTATAAGGCTACTAATTCGATTAGAGTTAGG

GAGAGGGGGAGTTTGGATGGGTAGAGAGGCCATCTATAACGTATTGGTGACTAGGCATGCAGTTATGATAGTATTCTTTT

TAGTTATACCAGTTTTTATGGGGGGGTTTGGTAACTGACTAATACCTGTTATATTAGGGTTGAGGGACATGGCCCTACCC

CGTTTAAATAATTTGAGGTTGATTTTACTACTTGCTAGGTTAGGGATTATGGGGGTATCATTACTGTTGGGTGGAGGTGG

GGCCGGTTGAACAATGTACCCACCCCTCATGTTGAGGGATTACAGGTCTGGGGTAGCTGTTGACTTAATAATTTTGGGGT

TGCATGTAGTGGGGTTGTCATCGATTTTAGGTTCAATTAATATTTTGGTTACTTGGGTGGCAGGGAGGAGCGTAGTATAT

AGTGTAGAGCAAGCCCCGTTATTTGTATGGGCTATAGTGACAACTGCGGGATTAGTGGTTTTGACGGTACCGGTTTTGGC

AGCAGCATTAACGATACTTTTGATGGACCGTAATTTGAACTCCAGGTTTTTTGA

>P.tereticollis_L3_Ponto-Caspian_Europe

GGTTTAATATATATTTTGGTTGGTGTGTGGGGAGGATTGATGGGGTTTTCTATGAGGCTACTAATTCGATTAGAGTTAGG

GAGAGGGGGAGTTTGGATGGGTAGAGAGGCCACCTATAACGTATTGGTGACTAGACATGCAGTTATGATAGTATTCTTTT

TAGTTATACCAGTTTTTATGGGGGGGTTTGGTAACTGACTAATACCTGTTATATTAGGGTTGAGGGACATGGCCCTACCC

CGTTTAAATAATTTGAGGTTGATTTTACTACTTGCTAGGTTAGGGATTATGGGGGTATCATTACTGTTGGGTGGAGGTGG

GGCCGGTTGAACAATGTACCCACCCCTCATGTTGAGGGATTACAGGTCTGGGGTAGCTGTTGATTTAATAATTTTGGGGT

TGCATGTAGTGGGGTTGTCATCGATTTTAGGTTCAATTAATATTTTGGTTACTTGGGTGGCAGGGAGGAGCGTAGTATAT

AGTGTAGAGCAAGCCCCGTTATTTGTATGGGCTATAGTGACAACTGCGGGATTAGTGGTTTTGACGGTACCGGTTTTGGC

AGCAGCATTAACGATACTTTTGATGGACCGTAATTTGAACTCCAGGTTTTTTGA

>P.tereticollis_L3_Western-Europe

GGTTTAATATATATTTTGGTTGGTGTGTGGGGAGGATTGATGGGGTTTTCTATAAGGCTACTAATTCGATTAGAGTTAGG

GAGAGGGGGAGTTTGGATGGGTAGAGAGGCCATCTATAACGTATTGGTGACTAGGCATGCAGTTATGATAGTATTCTTTT

TAGTTATACCAGTTTTTATGGGGGGGTTTGGTAACTGACTAATACCTGTTATATTAGGGTTGAGGGACATGGCCCTACCC

CGTTTAAATAATTTGAGGTTGATTTTACTACTTGCTAGGTTAGGGATTATGGGGGTATCATTACTGTTGGGTGGAGGTGG

GGCCGGTTGAACAATGTACCCACCCCTCATGTTGAGGGATTACAGGTCTGGGGTAGCTGTTGACTTAATAATTTTGGGGT

TGCATGTAGTGGGGTTGTCATCGATTTTAGGTTCAATTAATATTTTGGTTACTTGGGTGGCAGGGAGGAGCGTAGTATAT

AGTGTAGAGCAAGCCCCGTTATTTGTATGGGCTATAGTGACAACTGCGGGATTAGTGGTTTTGACGGTACCGGTTTTGGC

AGCAGCATTAACGATACTTTTGATGGACCGTAATTTGAACTCCAGGTTTTTTGA
