# Supplementary material for: Morphological and molecular data show no evidence of the proposed replacement of endemic Pomphorhynchus tereticollis by invasive P. laevis in salmonids in southern Germany
Source: PLoS One. 2020 Jun 16;15(6):e0234116. doi: 10.1371/journal.pone.0234116 (PMC7297375; doi:10.1371/journal.pone.0234116)
Supplement: S4 Table — (DOCX) [file pone.0234116.s005.docx]

**S5 Table. qPCR results supporting the morphological characterization**

| Species | Locality | Host | internal primer qPCR results (CT value) | | | Sanger sequencing PCR | |
| --- | --- | --- | --- | --- | --- | --- | --- |
|  |  |  | CT-L | CT-P1 | CT-P2 | COI | ITS |
| *P.tereticollis* | Nonnenbach, Kressbron | Bachforelle | negativ (25) | positive (15) | positive (15) | MT216159 |  |
| *P.tereticollis* | Georgenau | Bachforelle | negativ (25) | positive (14) | positive (14) | MT216155 |  |
| *P.tereticollis* | Nonnenbach, Kressbron | Bachforelle | negativ (25) | positive (11) | positive (11) | MT216158 |  |
| *P.tereticollis* | Nonnenbach, Kressbron | Bachforelle | negativ (25) | positive (13) | positive (13) | MT216165 |  |
| *P.tereticollis* | Nonnenbach, Kressbron | Bachforelle | negativ (25) | positive (16) | positive (16) | MT216154 |  |
| *P.tereticollis* | Nonnenbach, Kressbron | Chub | negativ (25) | positive (13) | positive (13) | MT216172 | MT216145 |
| *P.tereticollis* | Nonnenbach, Kressbron | Bachforelle | negativ (25) | positive (13) | positive (13) |  |  |
| *P.tereticollis* | Nonnenbach, Kressbron | Bachforelle | negativ (25) | positive (17) | positive (16) |  |  |
| *P.tereticollis* | Nonnenbach, Kressbron | Chub | negativ (25) | positive (15) | positive (14) |  |  |
| *P.tereticollis* | Nonnenbach, Kressbron | Chub | negativ (25) | positive (14) | positive (12) | MT216157 |  |
| *P.tereticollis* | Argen, Oberdorf | Chub | negativ (25) | positive (12) | positive (12) | MT216156 |  |
| *P.laevis* | Kinzig, Gengenbach | Chub | positive (9) | negativ (24) | negativ (20) |  |  |
| *P.laevis* | Kinzig, Gengenbach | Chub | positive (9) | negativ (23) | negativ (25) | MT216151 |  |
| *P.laevis* | Kinzig, Gengenbach | Chub | positive (10) | negativ (24) | negativ (20) | MT216152 | MT216138 |
| *P.tereticollis* | Brunnisach bei Hofen | Bachforelle | positive (17) | positive (14) | positive (13) | not passed | MT216148 |
| *P.tereticollis* | Schwarzach beim Mühlenholz | Bachforelle | negativ (25) | positive (15) | positive (14) |  |  |
| *P.tereticollis* | Schwarzach bei Untereschach | Bachforelle | negativ (25) | positive (15) | positive (14) |  |  |
| *P.tereticollis* | Lipbach bei Kluftern | Bachforelle | negativ (25) | positive (15) | positive (13) |  | MT216148 |
| *E.trutta* | Schwarzach bei Lindenmühle | Bachforelle | negativ (25) | negativ (25) | negativ (20) | MT216150 | MT216137 |
| *P.laevis* | Kanzach bei Dürmentingen | Bachforelle | positive (14) | negativ (25) | negativ (20) | MT216153 |  |
| *P.tereticollis* | Neckar bei Epfendorf | Aesche | negativ (25) | positive (14) | positive (12) | MT216169 | MT216143 |
| *P.tereticollis* | Neckar bei Bieringen | Aesche | negativ (25) | positive (14) | positive (13) | MT216168 | MT216142 |
| *P.tereticollis* | Ostrach bei Beizkofen | Bachforelle | negativ (25) | positive (17) | positive (16) | MT216162 | MT216140 |
| *P.tereticollis* | Wutach bei Ofteringen | Bachforelle | negativ (25) | positive (15) | positive (14) |  |  |
| *P.tereticollis* | Baierzer Rot bei Stetten | Aesche | negativ (25) | positive (16) | positive (14) | MT216167 |  |
| *P.tereticollis* | Baierzer Rot bei Stetten | Aesche | negativ (25) | positive (17) | positive (16) |  | MT216146 |
| *P.tereticollis* | Brunnisach bei Hofen | Bachforelle | negativ (25) | positive (13) | positive (12) |  |  |
| P.tereticollis | Schwarzach beim Mühlenholz | Bachforelle | negativ (25) | positive (13) | positive (12) | MT216160 | MT216139 |
| *P.tereticollis* | Schwarzach beim Mühlenholz | Bachforelle | negativ (25) | positive (16) | positive (16) |  |  |
| *P.tereticollis* | Lipbach bei Kluftern | Bachforelle | negativ (25) | positive (18) | positive (17) | MT216171 |  |
| *P.tereticollis* | Ostrach bei Beizkofen | Bachforelle | negativ (25) | positive (15) | positive (15) | MT216166 |  |
| *P.tereticollis* | Alb bei Beiertheim | Bachforelle | negativ (25) | positive (14) | positive (13) | MT216170 | MT216144 |
| *P.tereticollis* | Starzel bei Rangendingen | Bachforelle | negativ (25) | positive (14) | positive (13) |  |  |
| *P.tereticollis* | Starzel bei Friedrichstraße | Bachforelle | negativ (25) | positive (15) | positive (15) |  | not passed |
| *E.trutta* | Brenz bei Bergenweiler | Bachforelle | negativ (25) | negativ (25) | negativ (21) | MT216149 | MT216136 |
| *P.tereticollis* | Steinach bei Altneudorf | Bachforelle | negativ (25) | positive (17) | positive (16) | MT216161 |  |
| *P.tereticollis* | Schlücht bei Gutenburg | Bachforelle | negativ (25) | positive (13) | positive (13) |  |  |
| *P.tereticollis* | Würm bei Hausen | Bachforelle | negativ (25) | positive (14) | positive (13) | MT216164 | MT216141 |
| *P.tereticollis* | Forbach | Bachforelle | negativ (25) | positive (13) | positive (12) | MT216163 |  |
| *P.tereticollis* | Donau bei Geisingen | Bachforelle | negativ (25) | positive (13) | positive (12) |  |  |
| *P.tereticollis* | Wutach bei Eberfingen | Bachforelle | negativ (25) | positive (13) | positive (13) | not passed |  |
