# Supplementary material for: Morphological and molecular data show no evidence of the proposed replacement of endemic Pomphorhynchus tereticollis by invasive P. laevis in salmonids in southern Germany
Source: PLoS One. 2020 Jun 16;15(6):e0234116. doi: 10.1371/journal.pone.0234116 (PMC7297375; doi:10.1371/journal.pone.0234116)
Supplement: S1 Appendix — (DOC) [file pone.0234116.s006.doc]

**S6 Appendix statistical result**

**Standard least squares analysis in JMP**

**Response variable Sqrt(P_tereticollis)**

**Effect Summary**

| **Source** | **LogWorth** |  | **PValue** |
| --- | --- | --- | --- |
| Length | 2,831 |  | 0,00147 |
| Length*Host | 0,365 |  | 0,43170 |
| Waterbody | 0,234 |  | 0,58412 |
| Host | 0,180 |  | 0,65997 |

**Summary of Fit**

| RSquare | 0,341547 |
| --- | --- |
| RSquare Adj | 0,306585 |
| Root Mean Square Error | 0,515587 |
| Mean of Response | 1,628749 |
| Observations (or Sum Wgts) | 120 |

**Parameter Estimates**

| **Term** | **Estimate** | **Std Error** | **DFDen** | **t Ratio** | **Prob>|t|** |
| --- | --- | --- | --- | --- | --- |
| Intercept | 0,5991122 | 0,366629 | 112,5 | 1,63 | 0,1050 |
| Waterbody[Bodensee] | 0,1879693 | 0,177024 | 11,95 | 1,06 | 0,3093 |
| Waterbody[Danube] | 0,0515638 | 0,224761 | 11,23 | 0,23 | 0,8227 |
| Waterbody[Neckar] | -0,158241 | 0,164194 | 21,35 | -0,96 | 0,3460 |
| Length | 0,007471 | 0,002288 | 105,8 | 3,27 | 0,0015* |
| Host[grayling] | -0,080211 | 0,17974 | 21,02 | -0,45 | 0,6600 |
| (Length-118,464)* Host[grayling] | 0,0018051 | 0,002287 | 105,8 | 0,79 | 0,4317 |

**REML Variance Component Estimates**

| **Random Effect** | **Var Ratio** | **Var Component** | **Std Error** | **95% Lower** | **95% Upper** | **Wald p-Value** | **Pct of Total** |
| --- | --- | --- | --- | --- | --- | --- | --- |
| River | 0,3827378 | 0,101743 | 0,0591522 | -0,014193 | 0,2176792 | 0,0854 | 27,680 |
| Residual |  | 0,2658295 | 0,0381462 | 0,2044495 | 0,3598476 |  | 72,320 |
| Total |  | 0,3675725 | 0,0653064 | 0,2669184 | 0,5385188 |  | 100,000 |

-2 LogLikelihood = 229,2778614

Note: Total is the sum of the positive variance components.

Total including negative estimates = 0,3675725

**Fixed Effect Tests**

| **Source** | **Nparm** | **DF** | **DFDen** | **F Ratio** | **Prob > F** |
| --- | --- | --- | --- | --- | --- |
| Waterbody | 3 | 3 | 14,76 | 0,6693 | 0,5841 |
| Length | 1 | 1 | 105,8 | 10,6616 | 0,0015* |
| Host | 1 | 1 | 21,02 | 0,1991 | 0,6600 |
| Length*Host | 1 | 1 | 105,8 | 0,6230 | 0,4317 |

**Multiple Comparisons for Waterbody**

**Least Squares Means Estimates**

Standard errors are based on transformed response.

| **Waterbody2** | **Estimate** | **Std Error** | **DF** | **Lower 95%** | **Upper 95%** |
| --- | --- | --- | --- | --- | --- |
| Bodensee | 2,7959981 | 0,25924910 | 14,438 | 1,2491814 | 4,9576576 |
| Danube | 2,3584309 | 0,23689226 | 14,718 | 1,0608000 | 4,1676620 |
| Neckar | 1,7580477 | 0,22538431 | 24,229 | 0,7412792 | 3,2071512 |
| Rhine | 1,9680219 | 0,22431883 | 20,101 | 0,8743960 | 3,4992663 |

**Tukey HSD All Pairwise Comparisons**

Quantile = 2,88764, Adjusted DF = 14,8, Adjustment = Tukey-Kramer

Differences are based on transformed response.

**All Pairwise Differences**

| **Waterbody2** | **-Waterbody2** | **Difference** | **Std Error** | **t Ratio** | **Prob>|t|** | **Lower 95%** | **Upper 95%** |
| --- | --- | --- | --- | --- | --- | --- | --- |
| Bodensee | Danube | 0,136405 | 0,3525364 | 0,39 | 0,9796 | -0,881593 | 1,154404 |
| Bodensee | Neckar | 0,346210 | 0,2799162 | 1,24 | 0,6144 | -0,462087 | 1,154507 |
| Bodensee | Rhine | 0,269262 | 0,2323182 | 1,16 | 0,6605 | -0,401590 | 0,940113 |
| Danube | Neckar | 0,209804 | 0,3183817 | 0,66 | 0,9107 | -0,709567 | 1,129176 |
| Danube | Rhine | 0,132856 | 0,3281434 | 0,40 | 0,9767 | -0,814703 | 1,080416 |
| Neckar | Rhine | -0,076948 | 0,2436706 | -0,32 | 0,9886 | -0,780581 | 0,626685 |

**All Pairwise Comparisons Scatterplot**

Dataset

| Waterbody | River | Host | Host length (mm) | Number of P_terreticollis |
| --- | --- | --- | --- | --- |
| Rhine | Alb | grayling | 135 | 0 |
| Rhine | Alb | grayling | 160 | 0 |
| Danube | Baierzer Rot | grayling | 157 | 0 |
| Danube | Baierzer Rot | grayling | 140,5 | 2 |
| Danube | Baierzer Rot | grayling | 142,5 | 6 |
| Danube | Baierzer Rot | grayling | 145 | 4 |
| Danube | Baierzer Rot | grayling | 150 | 4 |
| Danube | Baierzer Rot | grayling | 150,5 | 0 |
| Danube | Baierzer Rot | grayling | 154 | 0 |
| Danube | Baierzer Rot | grayling | 157 | 0 |
| Danube | Baierzer Rot | grayling | 160 | 0 |
| Danube | Baierzer Rot | grayling | 169 | 0 |
| Danube | Baierzer Rot | grayling | 175 | 0 |
| Danube | Donau | grayling | 99 | 0 |
| Danube | Donau | grayling | 103,4 | 0 |
| Danube | Donau | grayling | 104 | 0 |
| Danube | Donau | grayling | 104 | 0 |
| Danube | Donau | grayling | 104,5 | 1 |
| Danube | Donau | grayling | 104,5 | 0 |
| Danube | Donau | grayling | 105 | 0 |
| Danube | Donau | grayling | 105,5 | 0 |
| Danube | Donau | grayling | 108,5 | 0 |
| Danube | Donau | grayling | 111 | 0 |
| Danube | Donau | grayling | 204,5 | 4 |
| Danube | Donau | grayling | 216,5 | 4 |
| Danube | Donau | grayling | 222 | 6 |
| Danube | Donau | grayling | 120 | 0 |
| Danube | Donau | grayling | 120,5 | 0 |
| Danube | Donau | grayling | 124 | 0 |
| Danube | Donau | grayling | 129,5 | 0 |
| Danube | Donau | grayling | 136,5 | 1 |
| Danube | Donau | grayling | 138 | 0 |
| Danube | Donau | grayling | 138,5 | 1 |
| Danube | Donau | grayling | 139 | 1 |
| Danube | Donau | grayling | 140 | 0 |
| Danube | Donau | grayling | 151 | 0 |
| Neckar | Neckar | grayling | 136,5 | 2 |
| Neckar | Neckar | grayling | 137,5 | 6 |
| Neckar | Neckar | grayling | 138,5 | 0 |
| Neckar | Neckar | grayling | 141,5 | 0 |
| Neckar | Neckar | grayling | 156,5 | 0 |
| Neckar | Neckar | grayling | 157,5 | 0 |
| Neckar | Neckar | grayling | 159,5 | 0 |
| Neckar | Neckar | grayling | 173 | 5 |
| Neckar | Neckar | grayling | 162,5 | 4 |
| Neckar | Neckar | grayling | 173,5 | 0 |
| Neckar | Neckar | grayling | 177,5 | 0 |
| Neckar | Neckar | grayling | 182,5 | 0 |
| Neckar | Neckar | grayling | 185 | 1 |
| Neckar | Neckar | grayling | 191 | 0 |
| Danube | Schwarzach | grayling | 117,5 | 0 |
| Danube | Schwarzach | grayling | 130 | 0 |
| Danube | Schwarzach | grayling | 157,5 | 0 |
| Rhine | Wutach | grayling | 67,5 | 0 |
| Rhine | Wutach | grayling | 70 | 0 |
| Rhine | Wutach | grayling | 70 | 0 |
| Rhine | Wutach | grayling | 70,5 | 0 |
| Rhine | Wutach | grayling | 71 | 0 |
| Rhine | Wutach | grayling | 71 | 0 |
| Rhine | Wutach | grayling | 72 | 0 |
| Rhine | Wutach | grayling | 72 | 0 |
| Rhine | Wutach | grayling | 73 | 0 |
| Rhine | Wutach | grayling | 75,5 | 0 |
| Rhine | Alb | brown trout | 85 | 0 |
| Rhine | Alb | brown trout | 95 | 0 |
| Rhine | Alb | brown trout | 110 | 3 |
| Rhine | Alb | brown trout | 112 | 0 |
| Rhine | Alb | brown trout | 115 | 1 |
| Rhine | Alb | brown trout | 115 | 1 |
| Rhine | Alb | brown trout | 115 | 0 |
| Rhine | Alb | brown trout | 120 | 0 |
| Rhine | Alb | brown trout | 125 | 0 |
| Rhine | Alb | brown trout | 140 | 1 |
| Bodensee | Argen | brown trout | 94 | 0 |
| Bodensee | Argen | brown trout | 95,5 | 0 |
| Bodensee | Argen | brown trout | 97,5 | 0 |
| Bodensee | Argen | brown trout | 100,5 | 0 |
| Bodensee | Argen | brown trout | 105 | 0 |
| Bodensee | Argen | brown trout | 107,5 | 0 |
| Bodensee | Argen | brown trout | 101 | 0 |
| Bodensee | Argen | brown trout | 106,5 | 0 |
| Bodensee | Argen | brown trout | 111 | 0 |
| Bodensee | Argen | brown trout | 114 | 0 |
| Bodensee | Argen | brown trout | 125,5 | 0 |
| Bodensee | Argen | brown trout | 129,3 | 0 |
| Bodensee | Argen | brown trout | 134,5 | 0 |
| Bodensee | Argen | brown trout | 145 | 0 |
| Bodensee | Argen | brown trout | 145 | 0 |
| Bodensee | Argen | brown trout | 164 | 0 |
| Danube | Baierzer Rot | brown trout | 123,5 | 0 |
| Danube | Baierzer Rot | brown trout | 95,5 | 0 |
| Danube | Baierzer Rot | brown trout | 102 | 0 |
| Danube | Baierzer Rot | brown trout | 107 | 0 |
| Danube | Bära | brown trout | 92 | 0 |
| Danube | Bära | brown trout | 95 | 0 |
| Danube | Bära | brown trout | 102,5 | 0 |
| Danube | Bära | brown trout | 109 | 0 |
| Danube | Bära | brown trout | 111,8 | 0 |
| Danube | Bära | brown trout | 118,5 | 0 |
| Danube | Bära | brown trout | 119 | 0 |
| Danube | Bära | brown trout | 127,5 | 0 |
| Danube | Blau | brown trout | 75,5 | 0 |
| Danube | Blau | brown trout | 94,5 | 0 |
| Danube | Blau | brown trout | 95 | 0 |
| Danube | Blau | brown trout | 97,5 | 0 |
| Danube | Blau | brown trout | 98,5 | 0 |
| Danube | Blau | brown trout | 100 | 0 |
| Danube | Blau | brown trout | 101 | 0 |
| Danube | Blau | brown trout | 101,5 | 0 |
| Danube | Blau | brown trout | 109,5 | 0 |
| Danube | Blau | brown trout | 112 | 0 |
| Danube | Brenz | brown trout | 85 | 0 |
| Danube | Brenz | brown trout | 129 | 0 |
| Danube | Brenz | brown trout | 135 | 0 |
| Danube | Brenz | brown trout | 153 | 0 |
| Danube | Brenz | brown trout | 155 | 0 |
| Danube | Brenz | brown trout | 103 | 0 |
| Danube | Brenz | brown trout | 106 | 0 |
| Danube | Brenz | brown trout | 114 | 0 |
| Danube | Brenz | brown trout | 115 | 0 |
| Danube | Brenz | brown trout | 118 | 0 |
| Danube | Brenz | brown trout | 123 | 0 |
| Danube | Brenz | brown trout | 127 | 0 |
| Danube | Brenz | brown trout | 130 | 0 |
| Danube | Brenz | brown trout | 140 | 0 |
| Danube | Brenz | brown trout | 150 | 0 |
| Bodensee | Brunnisach | brown trout | 76 | 0 |
| Bodensee | Brunnisach | brown trout | 83 | 4 |
| Bodensee | Brunnisach | brown trout | 85,5 | 0 |
| Bodensee | Brunnisach | brown trout | 89,4 | 3 |
| Bodensee | Brunnisach | brown trout | 92,5 | 3 |
| Bodensee | Brunnisach | brown trout | 92,5 | 0 |
| Bodensee | Brunnisach | brown trout | 95,5 | 5 |
| Bodensee | Brunnisach | brown trout | 96,5 | 7 |
| Bodensee | Brunnisach | brown trout | 100,5 | 2 |
| Bodensee | Brunnisach | brown trout | 110 | 0 |
| Rhine | Bühlot | brown trout | 73 | 4 |
| Rhine | Bühlot | brown trout | 77,5 | 4 |
| Rhine | Bühlot | brown trout | 83,5 | 0 |
| Rhine | Bühlot | brown trout | 86,5 | 2 |
| Rhine | Bühlot | brown trout | 94 | 0 |
| Rhine | Bühlot | brown trout | 99 | 0 |
| Rhine | Bühlot | brown trout | 118,5 | 0 |
| Rhine | Bühlot | brown trout | 119 | 6 |
| Rhine | Bühlot | brown trout | 129 | 6 |
| Rhine | Bühlot | brown trout | 129,5 | 4 |
| Rhine | Dreisam | brown trout | 61 | 0 |
| Rhine | Dreisam | brown trout | 62,5 | 0 |
| Rhine | Dreisam | brown trout | 63 | 0 |
| Rhine | Dreisam | brown trout | 63,5 | 0 |
| Rhine | Dreisam | brown trout | 73 | 0 |
| Rhine | Dreisam | brown trout | 73 | 0 |
| Rhine | Dreisam | brown trout | 73,5 | 0 |
| Rhine | Dreisam | brown trout | 83 | 0 |
| Rhine | Dreisam | brown trout | 126,5 | 0 |
| Rhine | Dreisam | brown trout | 146 | 0 |
| Rhine | Dreisam | brown trout | 154 | 0 |
| Rhine | Dreisam | brown trout | 161 | 0 |
| Rhine | Dreisam | brown trout | 62 | 0 |
| Rhine | Dreisam | brown trout | 64 | 0 |
| Rhine | Dreisam | brown trout | 66,5 | 0 |
| Rhine | Dreisam | brown trout | 69 | 0 |
| Rhine | Dreisam | brown trout | 75 | 0 |
| Rhine | Dreisam | brown trout | 81 | 0 |
| Rhine | Dreisam | brown trout | 135,5 | 0 |
| Rhine | Dreisam | brown trout | 150 | 0 |
| Rhine | Dreisam | brown trout | 155,5 | 1 |
| Neckar | Elz | brown trout | 95 | 0 |
| Neckar | Elz | brown trout | 145 | 0 |
| Neckar | Elz | brown trout | 161,5 | 0 |
| Neckar | Elz | brown trout | 162,2 | 0 |
| Neckar | Elz | brown trout | 162,8 | 0 |
| Neckar | Elz | brown trout | 164 | 0 |
| Neckar | Elz | brown trout | 166,6 | 0 |
| Neckar | Elz | brown trout | 174,5 | 0 |
| Neckar | Elz | brown trout | 100,5 | 0 |
| Neckar | Elz | brown trout | 106,3 | 0 |
| Neckar | Elz | brown trout | 106,5 | 0 |
| Neckar | Elz | brown trout | 113,2 | 0 |
| Neckar | Elz | brown trout | 113,8 | 0 |
| Neckar | Elz | brown trout | 131,5 | 0 |
| Rhine | Erlenbach (Harmersbach) | brown trout | 85 | 0 |
| Rhine | Erlenbach (Harmersbach) | brown trout | 90,5 | 0 |
| Rhine | Erlenbach (Harmersbach) | brown trout | 95,5 | 0 |
| Rhine | Erlenbach (Harmersbach) | brown trout | 100 | 0 |
| Rhine | Erlenbach (Harmersbach) | brown trout | 102 | 0 |
| Rhine | Erlenbach (Harmersbach) | brown trout | 112 | 0 |
| Rhine | Erlenbach (Harmersbach) | brown trout | 115 | 0 |
| Rhine | Erlenbach (Harmersbach) | brown trout | 117,5 | 0 |
| Rhine | Erlenbach (Harmersbach) | brown trout | 120,5 | 0 |
| Rhine | Erlenbach (Harmersbach) | brown trout | 123,5 | 0 |
| Neckar | Erms | brown trout | 72 | 0 |
| Neckar | Erms | brown trout | 79,5 | 0 |
| Neckar | Erms | brown trout | 82,5 | 0 |
| Neckar | Erms | brown trout | 88 | 1 |
| Neckar | Erms | brown trout | 88,5 | 0 |
| Neckar | Erms | brown trout | 89,5 | 0 |
| Neckar | Erms | brown trout | 91,5 | 0 |
| Neckar | Erms | brown trout | 94 | 0 |
| Neckar | Erms | brown trout | 97 | 0 |
| Neckar | Erms | brown trout | 104 | 0 |
| Neckar | Eyach | brown trout | 90 | 0 |
| Neckar | Eyach | brown trout | 125 | 0 |
| Neckar | Eyach | brown trout | 91 | 0 |
| Neckar | Eyach | brown trout | 126,5 | 0 |
| Neckar | Eyach | brown trout | 136 | 0 |
| Neckar | Eyach | brown trout | 142 | 0 |
| Neckar | Eyach | brown trout | 158 | 0 |
| Rhine | Forbach | brown trout | 107 | 0 |
| Rhine | Forbach | brown trout | 108 | 1 |
| Rhine | Forbach | brown trout | 150,5 | 2 |
| Rhine | Forbach | brown trout | 171 | 2 |
| Rhine | Forbach | brown trout | 171,5 | 4 |
| Rhine | Forbach | brown trout | 172 | 0 |
| Rhine | Forbach | brown trout | 175 | 1 |
| Rhine | Forbach | brown trout | 178,5 | 12 |
| Rhine | Forbach | brown trout | 186,5 | 2 |
| Bodensee | Haslach | brown trout | 74,5 | 0 |
| Bodensee | Haslach | brown trout | 79 | 0 |
| Bodensee | Haslach | brown trout | 80 | 0 |
| Bodensee | Haslach | brown trout | 80 | 0 |
| Bodensee | Haslach | brown trout | 83,5 | 0 |
| Bodensee | Haslach | brown trout | 87 | 0 |
| Bodensee | Haslach | brown trout | 88 | 0 |
| Bodensee | Haslach | brown trout | 88,5 | 0 |
| Bodensee | Haslach | brown trout | 105,5 | 0 |
| Bodensee | Haslach | brown trout | 124 | 0 |
| Bodensee | Haslach | brown trout | 76,5 | 0 |
| Bodensee | Haslach | brown trout | 80,5 | 0 |
| Bodensee | Haslach | brown trout | 90 | 0 |
| Bodensee | Haslach | brown trout | 92,5 | 0 |
| Bodensee | Haslach | brown trout | 99 | 0 |
| Bodensee | Haslach | brown trout | 100 | 0 |
| Bodensee | Haslach | brown trout | 134,5 | 0 |
| Bodensee | Haslach | brown trout | 135 | 0 |
| Bodensee | Haslach | brown trout | 135,5 | 0 |
| Bodensee | Haslach | brown trout | 155 | 0 |
| Rhine | Hauensteiner_Alb | brown trout | 102,5 | 0 |
| Rhine | Hauensteiner_Alb | brown trout | 103 | 0 |
| Rhine | Hauensteiner_Alb | brown trout | 105 | 0 |
| Rhine | Hauensteiner_Alb | brown trout | 106 | 1 |
| Rhine | Hauensteiner_Alb | brown trout | 66 | 0 |
| Rhine | Hauensteiner_Alb | brown trout | 67 | 0 |
| Rhine | Hauensteiner_Alb | brown trout | 68,5 | 0 |
| Rhine | Hauensteiner_Alb | brown trout | 74 | 0 |
| Rhine | Hauensteiner_Alb | brown trout | 74 | 0 |
| Rhine | Hauensteiner_Alb | brown trout | 75,5 | 0 |
| Rhine | Hauensteiner_Alb | brown trout | 81,5 | 0 |
| Rhine | Hauensteiner_Alb | brown trout | 81,5 | 0 |
| Rhine | Hauensteiner_Alb | brown trout | 82,5 | 0 |
| Rhine | Hauensteiner_Alb | brown trout | 85 | 0 |
| Rhine | Hauensteiner_Alb | brown trout | 88 | 0 |
| Rhine | Hauensteiner_Alb | brown trout | 112,5 | 0 |
| Rhine | Hauensteiner_Alb | brown trout | 113,5 | 0 |
| Rhine | Hauensteiner_Alb | brown trout | 114,5 | 0 |
| Rhine | Hauensteiner_Alb | brown trout | 128 | 0 |
| Rhine | Hauensteiner_Alb | brown trout | 147 | 0 |
| Rhine | Hauensteiner_Alb | brown trout | 150 | 0 |
| Rhine | Hauensteiner_Alb | brown trout | 150,5 | 0 |
| Rhine | Hauensteiner_Alb | brown trout | 153 | 0 |
| Rhine | Hauensteiner_Alb | brown trout | 158,5 | 0 |
| Rhine | Hauensteiner_Alb | brown trout | 160 | 0 |
| Rhine | Hauensteiner_Alb | brown trout | 86 | 0 |
| Rhine | Hauensteiner_Alb | brown trout | 86,5 | 0 |
| Rhine | Hauensteiner_Alb | brown trout | 88,5 | 0 |
| Rhine | Hauensteiner_Alb | brown trout | 88,5 | 0 |
| Rhine | Hauensteiner_Alb | brown trout | 89 | 0 |
| Rhine | Hauensteiner_Alb | brown trout | 93 | 0 |
| Rhine | Hauensteiner_Alb | brown trout | 100 | 1 |
| Rhine | Hauensteiner_Alb | brown trout | 101 | 0 |
| Rhine | Hauensteiner_Alb | brown trout | 105,5 | 0 |
| Rhine | Hauensteiner_Alb | brown trout | 108 | 0 |
| Rhine | Hauensteiner_Alb | brown trout | 109,5 | 0 |
| Rhine | Hauensteiner_Alb | brown trout | 110 | 1 |
| Danube | Kanzach | brown trout | 93 | 0 |
| Danube | Kanzach | brown trout | 98 | 0 |
| Danube | Kanzach | brown trout | 100 | 0 |
| Danube | Kanzach | brown trout | 110 | 0 |
| Danube | Kanzach | brown trout | 125 | 0 |
| Rhine | Kinzig | brown trout | 145 | 0 |
| Rhine | Kinzig | brown trout | 139 | 0 |
| Rhine | Kinzig | brown trout | 120 | 0 |
| Rhine | Kinzig | brown trout | 81,5 | 0 |
| Rhine | Kinzig | brown trout | 83 | 0 |
| Rhine | Kinzig | brown trout | 83 | 0 |
| Rhine | Kinzig | brown trout | 84 | 0 |
| Rhine | Kinzig | brown trout | 87,5 | 0 |
| Rhine | Kinzig | brown trout | 90 | 0 |
| Rhine | Kinzig | brown trout | 91 | 0 |
| Rhine | Kinzig | brown trout | 103,5 | 0 |
| Rhine | Kinzig | brown trout | 105 | 0 |
| Rhine | Kinzig | brown trout | 105 | 0 |
| Rhine | Kinzig | brown trout | 108,5 | 0 |
| Rhine | Kinzig | brown trout | 110,5 | 0 |
| Rhine | Kinzig | brown trout | 111,5 | 0 |
| Rhine | Kinzig | brown trout | 115 | 0 |
| Rhine | Kinzig | brown trout | 116,5 | 0 |
| Rhine | Kinzig | brown trout | 118,5 | 0 |
| Rhine | Kinzig | brown trout | 122,5 | 0 |
| Rhine | Kinzig | brown trout | 123 | 0 |
| Rhine | Kinzig | brown trout | 94,5 | 0 |
| Rhine | Kinzig | brown trout | 103,5 | 0 |
| Rhine | Kinzig | brown trout | 108,5 | 0 |
| Rhine | Kinzig | brown trout | 110,5 | 0 |
| Rhine | Kinzig | brown trout | 111,5 | 0 |
| Rhine | Kinzig | brown trout | 112 | 0 |
| Rhine | Kinzig | brown trout | 115 | 0 |
| Rhine | Kinzig | brown trout | 120 | 0 |
| Rhine | Kinzig | brown trout | 121 | 0 |
| Rhine | Kinzig | brown trout | 130 | 0 |
| Rhine | Kinzig | brown trout | 132 | 0 |
| Neckar | Körsch | brown trout | 88,5 | 0 |
| Neckar | Körsch | brown trout | 94,5 | 4 |
| Neckar | Körsch | brown trout | 97,5 | 0 |
| Neckar | Körsch | brown trout | 104,5 | 2 |
| Neckar | Körsch | brown trout | 113,5 | 0 |
| Danube | Lauchert | brown trout | 103 | 0 |
| Danube | Lauchert | brown trout | 107 | 0 |
| Danube | Lauchert | brown trout | 109,5 | 0 |
| Danube | Lauchert | brown trout | 110,5 | 0 |
| Danube | Lauchert | brown trout | 111,5 | 0 |
| Danube | Lauchert | brown trout | 112,5 | 0 |
| Danube | Lauchert | brown trout | 114 | 0 |
| Danube | Lauchert | brown trout | 114,5 | 0 |
| Danube | Lauchert | brown trout | 117,5 | 0 |
| Danube | Lauchert | brown trout | 126,5 | 0 |
| Danube | Lautracher Ach | brown trout | 84,5 | 0 |
| Danube | Lautracher Ach | brown trout | 85 | 0 |
| Danube | Lautracher Ach | brown trout | 86 | 0 |
| Danube | Lautracher Ach | brown trout | 89 | 0 |
| Danube | Lautracher Ach | brown trout | 89,5 | 0 |
| Danube | Lautracher Ach | brown trout | 91 | 0 |
| Danube | Lautracher Ach | brown trout | 96 | 0 |
| Danube | Lautracher Ach | brown trout | 100,5 | 0 |
| Danube | Lautracher Ach | brown trout | 107,5 | 0 |
| Danube | Lautracher Ach | brown trout | 115 | 0 |
| Danube | Lautracher Ach | brown trout | 121 | 0 |
| Bodensee | Lipbach | brown trout | 73,5 | 0 |
| Bodensee | Lipbach | brown trout | 79,5 | 4 |
| Bodensee | Lipbach | brown trout | 84,5 | 1 |
| Bodensee | Lipbach | brown trout | 86 | 0 |
| Bodensee | Lipbach | brown trout | 90,5 | 1 |
| Bodensee | Lipbach | brown trout | 91,5 | 1 |
| Bodensee | Lipbach | brown trout | 98 | 1 |
| Bodensee | Lipbach | brown trout | 101,5 | 2 |
| Bodensee | Lipbach | brown trout | 101,5 | 0 |
| Bodensee | Lipbach | brown trout | 102,5 | 2 |
| Rhine | Murg | brown trout | 70 | 0 |
| Rhine | Murg | brown trout | 92 | 0 |
| Rhine | Murg | brown trout | 97 | 0 |
| Rhine | Murg | brown trout | 102 | 0 |
| Rhine | Murg | brown trout | 102 | 0 |
| Rhine | Murg | brown trout | 109 | 0 |
| Rhine | Murg | brown trout | 120 | 0 |
| Rhine | Murg | brown trout | 131 | 0 |
| Rhine | Murg | brown trout | 137 | 0 |
| Rhine | Murg | brown trout | 154 | 0 |
| Neckar | Nagold | brown trout | 103 | 0 |
| Neckar | Nagold | brown trout | 108 | 0 |
| Neckar | Nagold | brown trout | 115 | 0 |
| Neckar | Nagold | brown trout | 117 | 0 |
| Neckar | Nagold | brown trout | 120 | 0 |
| Neckar | Neckar | brown trout | 73 | 0 |
| Neckar | Neckar | brown trout | 76,5 | 0 |
| Neckar | Neckar | brown trout | 80,5 | 0 |
| Neckar | Neckar | brown trout | 86,5 | 0 |
| Neckar | Neckar | brown trout | 94 | 0 |
| Neckar | Neckar | brown trout | 98,5 | 0 |
| Neckar | Neckar | brown trout | 130 | 0 |
| Bodensee | Nonnenbach | brown trout | 61 | 5 |
| Bodensee | Nonnenbach | brown trout | 64 | 0 |
| Bodensee | Nonnenbach | brown trout | 65 | 1 |
| Bodensee | Nonnenbach | brown trout | 70 | 3 |
| Bodensee | Nonnenbach | brown trout | 70 | 2 |
| Bodensee | Nonnenbach | brown trout | 71 | 1 |
| Bodensee | Nonnenbach | brown trout | 73 | 2 |
| Bodensee | Nonnenbach | brown trout | 73 | 1 |
| Bodensee | Nonnenbach | brown trout | 74 | 2 |
| Bodensee | Nonnenbach | brown trout | 75 | 4 |
| Bodensee | Nonnenbach | brown trout | 75 | 0 |
| Bodensee | Nonnenbach | brown trout | 84 | 2 |
| Bodensee | Nonnenbach | brown trout | 99 | 3 |
| Bodensee | Nonnenbach | brown trout | 102,5 | 3 |
| Bodensee | Nonnenbach | brown trout | 112 | 5 |
| Bodensee | Nonnenbach | brown trout | 121 | 3 |
| Bodensee | Nonnenbach | brown trout | 125,5 | 0 |
| Bodensee | Nonnenbach | brown trout | 141 | 9 |
| Bodensee | Nonnenbach | brown trout | 155 | 15 |
| Bodensee | Nonnenbach | brown trout | 170 | 10 |
| Bodensee | Nonnenbach | brown trout | 177 | 6 |
| Bodensee | Nonnenbach | brown trout | 183 | 5 |
| Danube | Ostrach | brown trout | 72,5 | 0 |
| Danube | Ostrach | brown trout | 82,5 | 3 |
| Danube | Ostrach | brown trout | 99 | 5 |
| Danube | Ostrach | brown trout | 106,5 | 3 |
| Danube | Ostrach | brown trout | 107,5 | 1 |
| Danube | Ostrach | brown trout | 113,5 | 4 |
| Danube | Ostrach | brown trout | 116 | 6 |
| Danube | Ostrach | brown trout | 125 | 2 |
| Danube | Ostrach | brown trout | 126 | 3 |
| Danube | Ostrach | brown trout | 128 | 2 |
| Neckar | Rems | brown trout | 74,5 | 0 |
| Neckar | Rems | brown trout | 88,5 | 0 |
| Neckar | Rems | brown trout | 91,5 | 0 |
| Neckar | Rems | brown trout | 95 | 0 |
| Neckar | Rems | brown trout | 95 | 0 |
| Neckar | Rems | brown trout | 96 | 0 |
| Neckar | Rems | brown trout | 98,5 | 0 |
| Neckar | Rems | brown trout | 99 | 0 |
| Neckar | Rems | brown trout | 104,5 | 0 |
| Neckar | Rems | brown trout | 108 | 0 |
| Rhine | Rench | brown trout | 76,75 | 0 |
| Rhine | Rench | brown trout | 81 | 0 |
| Rhine | Rench | brown trout | 85,5 | 0 |
| Rhine | Rench | brown trout | 88,5 | 0 |
| Rhine | Rench | brown trout | 97,5 | 0 |
| Rhine | Rench | brown trout | 102,5 | 0 |
| Rhine | Rench | brown trout | 103,5 | 0 |
| Rhine | Rench | brown trout | 108,5 | 0 |
| Rhine | Rench | brown trout | 109,5 | 0 |
| Rhine | Rench | brown trout | 115 | 0 |
| Bodensee | Saubach | brown trout | 93 | 0 |
| Bodensee | Saubach | brown trout | 100 | 0 |
| Bodensee | Saubach | brown trout | 100 | 0 |
| Bodensee | Saubach | brown trout | 102 | 0 |
| Bodensee | Saubach | brown trout | 111 | 0 |
| Bodensee | Saubach | brown trout | 115 | 0 |
| Bodensee | Saubach | brown trout | 118 | 0 |
| Neckar | Schefflenz | brown trout | 64,1 | 0 |
| Neckar | Schefflenz | brown trout | 78 | 0 |
| Neckar | Schefflenz | brown trout | 79,5 | 0 |
| Neckar | Schefflenz | brown trout | 80 | 0 |
| Neckar | Schefflenz | brown trout | 84,5 | 0 |
| Neckar | Schefflenz | brown trout | 87,1 | 0 |
| Neckar | Schefflenz | brown trout | 92,9 | 0 |
| Neckar | Schefflenz | brown trout | 101,5 | 0 |
| Neckar | Schefflenz | brown trout | 102 | 0 |
| Neckar | Schefflenz | brown trout | 109,5 | 0 |
| Neckar | Schefflenz | brown trout | 183,5 | 0 |
| Neckar | Schefflenz | brown trout | 191,5 | 0 |
| Rhine | Schlücht | brown trout | 108 | 0 |
| Rhine | Schlücht | brown trout | 122 | 0 |
| Rhine | Schlücht | brown trout | 142 | 0 |
| Rhine | Schlücht | brown trout | 160 | 1 |
| Rhine | Schlücht | brown trout | 162 | 0 |
| Rhine | Schlücht | brown trout | 163 | 0 |
| Rhine | Schlücht | brown trout | 165 | 0 |
| Rhine | Schlücht | brown trout | 172 | 0 |
| Rhine | Schlücht | brown trout | 178 | 2 |
| Rhine | Schlücht | brown trout | 185 | 1 |
| Rhine | Schutter | brown trout | 83 | 3 |
| Rhine | Schutter | brown trout | 96,5 | 1 |
| Rhine | Schutter | brown trout | 99 | 4 |
| Rhine | Schutter | brown trout | 107,5 | 1 |
| Rhine | Schutter | brown trout | 111 | 7 |
| Rhine | Schutter | brown trout | 112 | 0 |
| Rhine | Schutter | brown trout | 118 | 7 |
| Rhine | Schutter | brown trout | 118,5 | 4 |
| Rhine | Schutter | brown trout | 119,5 | 2 |
| Rhine | Schutter | brown trout | 121,5 | 5 |
| Danube | Schwarzach | brown trout | 87,5 | 0 |
| Danube | Schwarzach | brown trout | 107,7 | 0 |
| Danube | Schwarzach | brown trout | 109,8 | 0 |
| Danube | Schwarzach | brown trout | 112 | 0 |
| Danube | Schwarzach | brown trout | 117,6 | 0 |
| Danube | Schwarzach | brown trout | 118,5 | 0 |
| Danube | Schwarzach | brown trout | 124 | 0 |
| Danube | Schwarzach | brown trout | 133 | 0 |
| Bodensee | Schwarzach | brown trout | 62,5 | 0 |
| Bodensee | Schwarzach | brown trout | 73,5 | 0 |
| Bodensee | Schwarzach | brown trout | 82 | 0 |
| Bodensee | Schwarzach | brown trout | 94 | 2 |
| Bodensee | Schwarzach | brown trout | 96 | 0 |
| Bodensee | Schwarzach | brown trout | 104 | 0 |
| Bodensee | Schwarzach | brown trout | 105 | 0 |
| Bodensee | Schwarzach | brown trout | 108 | 0 |
| Bodensee | Schwarzach | brown trout | 109,5 | 0 |
| Bodensee | Schwarzach | brown trout | 129 | 1 |
| Bodensee | Schwarzach | brown trout | 86 | 0 |
| Bodensee | Schwarzach | brown trout | 88,5 | 0 |
| Bodensee | Schwarzach | brown trout | 92 | 0 |
| Bodensee | Schwarzach | brown trout | 97,5 | 2 |
| Bodensee | Schwarzach | brown trout | 97,5 | 0 |
| Bodensee | Schwarzach | brown trout | 101,5 | 1 |
| Bodensee | Schwarzach | brown trout | 105,5 | 0 |
| Bodensee | Schwarzach | brown trout | 106,5 | 2 |
| Bodensee | Schwarzach | brown trout | 109 | 4 |
| Bodensee | Schwarzach | brown trout | 117,8 | 2 |
| Neckar | Seltenbach | brown trout | 77 | 0 |
| Neckar | Seltenbach | brown trout | 86,5 | 0 |
| Neckar | Seltenbach | brown trout | 91 | 0 |
| Neckar | Seltenbach | brown trout | 94 | 0 |
| Neckar | Seltenbach | brown trout | 97,5 | 0 |
| Neckar | Seltenbach | brown trout | 100,5 | 0 |
| Neckar | Seltenbach | brown trout | 101,5 | 0 |
| Neckar | Seltenbach | brown trout | 102,5 | 0 |
| Neckar | Seltenbach | brown trout | 103,5 | 0 |
| Neckar | Seltenbach | brown trout | 121,5 | 0 |
| Rhine | Starzel | brown trout | 80 | 3 |
| Rhine | Starzel | brown trout | 80 | 0 |
| Rhine | Starzel | brown trout | 85 | 3 |
| Rhine | Starzel | brown trout | 106 | 3 |
| Rhine | Starzel | brown trout | 142 | 2 |
| Neckar | Starzel | brown trout | 124 | 0 |
| Neckar | Starzel | brown trout | 217 | 2 |
| Neckar | Starzel | brown trout | 100 | 0 |
| Neckar | Starzel | brown trout | 105 | 0 |
| Neckar | Starzel | brown trout | 122 | 0 |
| Neckar | Starzel | brown trout | 125 | 0 |
| Neckar | Starzel | brown trout | 68 | 0 |
| Neckar | Starzel | brown trout | 76 | 0 |
| Neckar | Starzel | brown trout | 87 | 0 |
| Neckar | Starzel | brown trout | 88,5 | 0 |
| Neckar | Starzel | brown trout | 94 | 0 |
| Neckar | Starzel | brown trout | 95 | 0 |
| Neckar | Starzel | brown trout | 97 | 0 |
| Neckar | Starzel | brown trout | 98,5 | 0 |
| Rhine | Steina | brown trout | 120 | 0 |
| Rhine | Steina | brown trout | 135 | 0 |
| Rhine | Steina | brown trout | 142 | 0 |
| Rhine | Steina | brown trout | 145 | 0 |
| Rhine | Steina | brown trout | 162 | 0 |
| Rhine | Steina | brown trout | 192 | 0 |
| Neckar | Steinach | brown trout | 89 | 4 |
| Neckar | Steinach | brown trout | 95 | 3 |
| Neckar | Steinach | brown trout | 96 | 1 |
| Neckar | Steinach | brown trout | 97 | 3 |
| Neckar | Steinach | brown trout | 97 | 0 |
| Neckar | Steinach | brown trout | 102 | 0 |
| Neckar | Steinach | brown trout | 105 | 1 |
| Neckar | Steinach | brown trout | 118 | 0 |
| Neckar | Steinach | brown trout | 138 | 0 |
| Neckar | Steinach | brown trout | 156 | 1 |
| Rhine | Wehra | brown trout | 93,5 | 1 |
| Rhine | Wehra | brown trout | 95 | 0 |
| Rhine | Wehra | brown trout | 99,5 | 0 |
| Rhine | Wehra | brown trout | 102 | 1 |
| Rhine | Wehra | brown trout | 104 | 2 |
| Rhine | Wehra | brown trout | 104,5 | 0 |
| Rhine | Wehra | brown trout | 107,5 | 4 |
| Rhine | Wehra | brown trout | 112,5 | 3 |
| Rhine | Wehra | brown trout | 120 | 1 |
| Rhine | Wehra | brown trout | 121,5 | 0 |
| Rhine | Wehra | brown trout | 122 | 0 |
| Rhine | Wehra | brown trout | 126 | 1 |
| Rhine | Wiese | brown trout | 76 | 0 |
| Rhine | Wiese | brown trout | 88 | 0 |
| Rhine | Wiese | brown trout | 90,5 | 0 |
| Rhine | Wiese | brown trout | 91,5 | 0 |
| Rhine | Wiese | brown trout | 100 | 0 |
| Rhine | Wiese | brown trout | 107,5 | 0 |
| Rhine | Wiese | brown trout | 108,5 | 0 |
| Rhine | Wiese | brown trout | 111,5 | 0 |
| Rhine | Wiese | brown trout | 121 | 0 |
| Rhine | Wiese | brown trout | 63,5 | 0 |
| Rhine | Wiese | brown trout | 64,5 | 0 |
| Rhine | Wiese | brown trout | 64,5 | 0 |
| Rhine | Wiese | brown trout | 70 | 0 |
| Rhine | Wiese | brown trout | 70,5 | 0 |
| Rhine | Wiese | brown trout | 74 | 0 |
| Rhine | Wiese | brown trout | 74,5 | 0 |
| Rhine | Wiese | brown trout | 76,5 | 0 |
| Rhine | Wiese | brown trout | 80 | 0 |
| Rhine | Wiese | brown trout | 81 | 0 |
| Rhine | Wiese | brown trout | 104 | 0 |
| Rhine | Wiese | brown trout | 118,5 | 0 |
| Rhine | Wiese | brown trout | 66 | 0 |
| Rhine | Wiese | brown trout | 67 | 0 |
| Rhine | Wiese | brown trout | 72 | 0 |
| Rhine | Wiese | brown trout | 78,5 | 0 |
| Rhine | Wiese | brown trout | 82 | 0 |
| Rhine | Wiese | brown trout | 82 | 0 |
| Rhine | Wiese | brown trout | 87 | 0 |
| Rhine | Wiese | brown trout | 90,5 | 0 |
| Rhine | Wiese | brown trout | 91,5 | 0 |
| Rhine | Wiese | brown trout | 97 | 0 |
| Bodensee | Wolfegger Ach | brown trout | 69 | 0 |
| Bodensee | Wolfegger Ach | brown trout | 81 | 0 |
| Bodensee | Wolfegger Ach | brown trout | 83 | 0 |
| Bodensee | Wolfegger Ach | brown trout | 87,5 | 0 |
| Bodensee | Wolfegger Ach | brown trout | 90,5 | 0 |
| Bodensee | Wolfegger Ach | brown trout | 91,5 | 0 |
| Bodensee | Wolfegger Ach | brown trout | 94,5 | 0 |
| Bodensee | Wolfegger Ach | brown trout | 96 | 0 |
| Bodensee | Wolfegger Ach | brown trout | 98,5 | 0 |
| Bodensee | Wolfegger Ach | brown trout | 98,5 | 0 |
| Neckar | Würm | brown trout | 93,5 | 0 |
| Neckar | Würm | brown trout | 96,5 | 0 |
| Neckar | Würm | brown trout | 98,5 | 0 |
| Neckar | Würm | brown trout | 104 | 0 |
| Neckar | Würm | brown trout | 105,5 | 1 |
| Neckar | Würm | brown trout | 107 | 0 |
| Neckar | Würm | brown trout | 110 | 2 |
| Neckar | Würm | brown trout | 110 | 0 |
| Neckar | Würm | brown trout | 111 | 0 |
| Neckar | Würm | brown trout | 120 | 0 |
| Rhine | Wutach | brown trout | 82,5 | 0 |
| Rhine | Wutach | brown trout | 83 | 0 |
| Rhine | Wutach | brown trout | 87 | 0 |
| Rhine | Wutach | brown trout | 87,5 | 0 |
| Rhine | Wutach | brown trout | 91,5 | 0 |
| Rhine | Wutach | brown trout | 97 | 0 |
| Rhine | Wutach | brown trout | 99,5 | 0 |
| Rhine | Wutach | brown trout | 111,5 | 0 |
| Rhine | Wutach | brown trout | 112,5 | 0 |
| Rhine | Wutach | brown trout | 119,5 | 0 |
| Rhine | Wutach | brown trout | 120,5 | 0 |
| Rhine | Wutach | brown trout | 123,5 | 0 |
| Rhine | Wutach | brown trout | 127 | 0 |
| Rhine | Wutach | brown trout | 60,5 | 0 |
| Rhine | Wutach | brown trout | 62,5 | 0 |
| Rhine | Wutach | brown trout | 72,5 | 0 |
| Rhine | Wutach | brown trout | 76,5 | 0 |
| Rhine | Wutach | brown trout | 100 | 0 |
| Rhine | Wutach | brown trout | 104 | 0 |
| Rhine | Wutach | brown trout | 105,5 | 0 |
| Rhine | Wutach | brown trout | 111 | 0 |
| Rhine | Wutach | brown trout | 116 | 0 |
| Rhine | Wutach | brown trout | 142,5 | 0 |
| Rhine | Wutach | brown trout | 155,5 | 0 |
| Rhine | Wutach | brown trout | 184 | 0 |
| Rhine | Wutach | brown trout | 206,5 | 0 |
| Rhine | Wutach | brown trout | 209,5 | 0 |
| Rhine | Wutach | brown trout | 224 | 0 |
| Rhine | Wutach | brown trout | 115 | 0 |
| Rhine | Wutach | brown trout | 115 | 0 |
| Rhine | Wutach | brown trout | 125 | 0 |
| Rhine | Wutach | brown trout | 130 | 0 |
| Rhine | Wutach | brown trout | 135 | 0 |
| Rhine | Wutach | brown trout | 140 | 0 |
| Rhine | Wutach | brown trout | 145 | 0 |
| Rhine | Wutach | brown trout | 150 | 0 |
| Rhine | Wutach | brown trout | 160 | 0 |
| Rhine | Wutach | brown trout | 160 | 0 |
| Rhine | Wutach | brown trout | 225 | 0 |
| Rhine | Wutach | brown trout | 240 | 0 |
| Rhine | Wutach | brown trout | 135 | 0 |
| Rhine | Wutach | brown trout | 140 | 0 |
| Rhine | Wutach | brown trout | 145 | 0 |
| Rhine | Wutach | brown trout | 148 | 0 |
| Rhine | Wutach | brown trout | 150 | 0 |
| Rhine | Wutach | brown trout | 155 | 0 |
| Rhine | Wutach | brown trout | 155 | 0 |
| Rhine | Wutach | brown trout | 155 | 0 |
| Rhine | Wutach | brown trout | 160 | 0 |
| Rhine | Wutach | brown trout | 165 | 0 |
